# FastMI-HGNet: A Two-Stream Heterogeneous Graph Neural Network for Multi-Omics Disease Classification

**DOI:** 10.3390/genes17080865

**Published:** 2026-07-24

**Authors:** Xufeng Fu, Bipeng Lai, Rongling He, Xiuji Huang, Qin Jiang, Hui Li

**Affiliations:** Department of Respiratory and Critical Care Medicine, The Seventh Affiliated Hospital of Sun Yat-sen University, No. 628 Zhenyuan Road, Guangming District, Shenzhen 518107, China; fuxufeng@sysush.com (X.F.); laibipeng@sysush.com (B.L.); herongling@sysush.com (R.H.); huangxiuji@sysush.com (X.H.); jiangqin@sysush.com (Q.J.)

**Keywords:** multi-omics integration, disease classification, heterogeneous graph neural network, mutual information, biomarker prioritization, bioinformatics

## Abstract

**Background/Objectives**: Multi-omics datasets are increasingly used for disease classification, but differences in scale, distribution, and resolution across omics layers complicate their integration. Conventional fusion approaches may overlook nonlinear cross-omics dependencies and structured sample–feature relationships. Here, we propose FastMI-HGNet, a two-stream heterogeneous graph neural network for multi-omics disease classification. **Methods**: The framework uses fast mutual information (FastMI) to construct dependency edge priors for a heterogeneous graph that connects sample and feature nodes. A Transformer-based data stream captures vector-level feature interactions, while a graph attention stream models structural dependencies among samples and molecular features. An uncertainty-aware ensemble further improves stability under small-sample and noisy multi-omics settings. **Results**: Evaluated on five public multi-omics benchmarks—ROSMAP, LGG, BRCA, and the more challenging COAD tumor-stage classification task, together with KIPAN as a ceiling-level proof-of-concept benchmark—FastMI-HGNet achieved competitive classification performance while supporting interpretable biomarker prioritization. In BRCA, SHAP-based analysis highlighted model-prioritized genes such as FOXC1 and SOX10. **Conclusions**: FastMI-HGNet supports interpretable multi-omics disease classification and biomarker prioritization.

## 1. Introduction

Disease classification based on multi-omics profiles is an important task in precision medicine, with cancer molecular subtyping representing one of its most biologically relevant applications. Recent advances in high-throughput technologies have facilitated the collection of multi-omics data (e.g., genomics, epigenomics, transcriptomics, proteomics), offering unprecedented opportunities to dissect the molecular mechanisms underlying complex diseases and cancers [1]. By leveraging complementary biological information across different omics layers, multi-omics fusion can enhance the accuracy and interpretability of disease classification [2]. For example, integrating genomic, transcriptomic, and digital pathology data has led to high accuracy in predicting breast cancer therapy response [3].

Despite these advantages, the inherent heterogeneity of multi-omics data—manifested in differences in data distributions, high dimensionality, and limited sample sizes—poses substantial challenges to data integration [4]. These properties make it difficult for traditional statistical methods to capture the complex, nonlinear interactions among multiple omics layers, often resulting in suboptimal performance. Deep learning (DL) methods, by contrast, have demonstrated remarkable capabilities in automatically extracting high-level representations from large-scale, high-dimensional data, making it possible to better model cross-omics dependencies [5].

Early DL-based frameworks for multi-omics integration primarily employed unsupervised representation learning. For instance, Chaudhary et al. utilized stacked denoising autoencoders to stratify hepatocellular carcinoma, successfully identifying subgroups with distinct survival outcomes [5]. Subsequently, supervised architectures were introduced to improve classification performance, such as omic-specific encoders for breast cancer subtyping [6] and ensemble frameworks for survival prediction [7]. Variational autoencoders were also leveraged to learn nonlinear embeddings and reveal meaningful biological signals [8]. However, these earlier DL methods often merely concatenated features across omics, neglecting their unique distributions and interdependencies.

To better capture cross-omics interactions, researchers have explored attention mechanisms and biologically informed priors. Attention-based models like SADLN [9] and DMOIT [10] dynamically weight omics features, thereby emphasizing crucial cross-omic signals and improving classification performance. Other strategies incorporate known biological pathways [11] or multi-task learning [12] to enhance predictive power and interpretability. Nevertheless, most of these methods operate in a purely vectorized space, overlooking the inherent network structure of biological data. Such network structure can arise from multiple sources, including gene–gene co-expression networks, protein–protein interaction networks, and genomic regulatory relationships, all of which can provide valuable information on molecular connectivity and functional similarity.

Graph-based approaches offer a more structured paradigm for multi-omics data integration by representing samples and/or molecular features as nodes and their relationships (e.g., patient similarity, gene co-expression) as edges. Patient similarity networks (PSNs), for instance, have shown efficacy in multi-omics fusion and improved interpretability [13]. Graph neural networks (GNNs), particularly graph convolutional networks (GCNs), enable information propagation across connected nodes, capturing dependencies that flat feature-based methods may miss [14]. Notable examples include MOGONET [15], which constructs omic-specific graphs for feature embedding but does not fully account for inter-omic feature correlations, and MoGCN [16], which builds patient similarity graphs for cancer subtype prediction yet lacks a mechanism to explicitly model sample–feature interactions.

Although graph-based integration strategies have advanced the field, they still face several limitations. Many rely on homogeneous graphs (either samples or features only), thereby failing to capture the shared and distinct information across different omics layers, which in turn leads to suboptimal fusion performance. Moreover, methods that do consider multiple omics often treat them as separate inputs rather than seamlessly integrating them to exploit cross-omics synergy. For example, MOGONET focuses on individual omics graphs separately and then fuses the results, not fully exploring omic-level interactions. Likewise, MoGCN constructs patient graphs but overlooks direct sample–feature edges, limiting the model’s ability to identify crucial molecular signatures. Consequently, essential associations between specific genomic variations and patient outcomes may be missed. Additionally, practical constraints such as high dimensionality and small sample sizes constrain graph model complexity, often necessitating shallow architectures that cannot capture long-range dependencies [17].

Recent progress in trustworthy multimodal learning addresses some of these challenges through dynamic fusion mechanisms. Han et al. [18] presented a multimodal dynamic framework that adaptively weighs both feature- and modality-level contributions to enhance reliability. Zheng et al. [19] developed MLCLNet, which integrates confidence learning at both feature and label levels to improve robustness. Inspired by these developments, we propose extending dynamic and confidence-aware strategies to the multi-omics domain, incorporating mutual-information-based dependency priors and graph-based representations to improve disease classification reliability and interpretability.

In this work, we introduce **FastMI-HGNet**, a two-stream heterogeneous graph framework for multi-omics disease classification. First, fast mutual information (FastMI) is used to identify strong nonlinear dependency patterns among omics features. These dependencies are incorporated as edge priors to construct a heterogeneous graph containing both sample and feature nodes. The resulting graph captures sample–sample similarities, sample–feature associations, and feature–feature dependency patterns across omics layers. We then develop a dual-stream fusion architecture: (1) a *data stream*, which applies a Transformer encoder to capture vectorized omic feature interactions, and (2) a *graph stream*, which employs a graph attention network (GAT) to model structural dependencies on the FastMI-guided heterogeneous graph. Finally, an uncertainty-aware ensemble strategy dynamically weights predictions from multiple dual-stream models, improving stability under limited-sample and noisy multi-omics settings. We emphasize that the individual building blocks—mutual-information estimation, Transformer encoders, graph attention networks, and uncertainty-aware ensembling—are not novel in isolation; the novelty of FastMI-HGNet lies in *unifying* them into a single heterogeneous-graph framework in which FastMI-derived cross-omics dependency edges couple the vector-level (data-stream) and graph-level (graph-stream) representations.

Experiments on five public multi-omics benchmarks—ROSMAP, LGG, BRCA, and the more challenging COAD tumor-stage classification task, together with KIPAN as a ceiling-level benchmark—show that FastMI-HGNet achieves competitive accuracy, F1-score, and AUC compared with existing integration methods, while providing interpretable feature-level evidence for biomarker prioritization.

## 2. Materials and Methods

### 2.1. Methodological Overview

**Figure 1:**
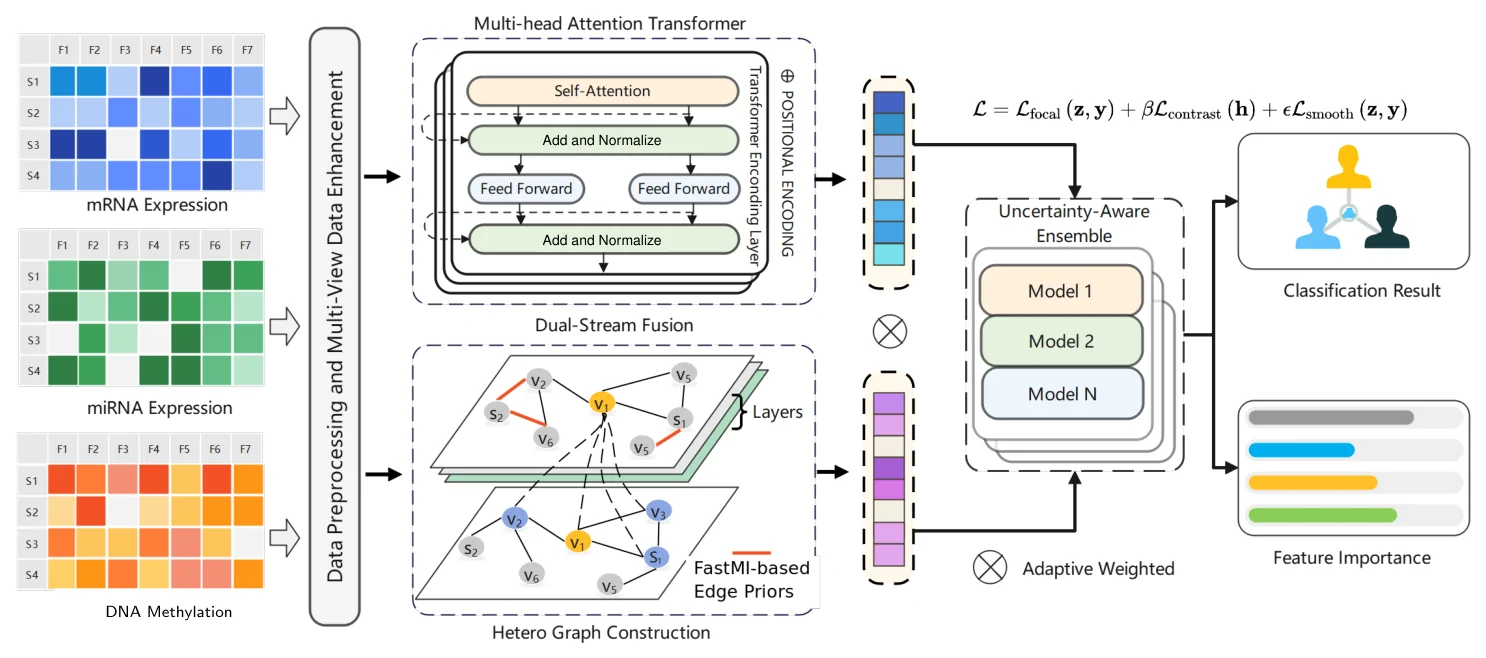
Overview of the proposed FastMI-guided heterogeneous graph-based dual-stream fusion framework.

The integration of multi-omics data provides valuable insights into complex diseases by capturing complementary molecular interactions. To address challenges such as high dimensionality, noise sensitivity, and heterogeneity across omics layers, we propose a FastMI-guided heterogeneous graph-based dual-stream fusion framework, as illustrated in Figure 1.

Our framework comprises four main components:

First, we perform data preprocessing on multi-omics datasets from public repositories, including sample alignment, missing value imputation, normalization, and controlled data perturbations to enhance model robustness.

Second, we construct a heterogeneous graph that integrates sample nodes and omics-specific feature nodes. The graph edges are established based on: (1) sample–sample similarities, (2) feature–sample associations, and (3) strong nonlinear statistical dependencies between features inferred using a k-nearest neighbor-based fast mutual information estimation (FastMI) method.

Third, we design a dual-stream fusion model that processes data through parallel pathways: a Transformer-based data stream that captures high-level feature interactions across omics layers, and a graph attention network (GAT) that models structural dependencies in the heterogeneous graph. The outputs from both streams are adaptively weighted to obtain the final representation.

Finally, we implement an uncertainty-aware ensemble learning strategy where multiple dual-stream models with diverse configurations are trained independently, and their predictions are dynamically weighted based on uncertainty measures, enhancing model reliability and enabling identification of low-confidence samples.

The subsequent sections detail the specific implementations of each component and present comprehensive experimental evaluations.

To facilitate reproducibility, Algorithm 1 summarizes the end-to-end FastMI-HGNet pipeline, integrating preprocessing, FastMI-based dependency edge construction, heterogeneous graph construction, dual-stream fusion, and the uncertainty-aware ensemble.
**Algorithm 1** FastMI-HGNet end-to-end training and inference pipeline.**Require:** Multi-omics data ${\left\{X^{\left(m\right)}\right\}}_{m=1}^{3}$ (mRNA, miRNA, methylation), labels y, ensemble size *M*, FastMI threshold τ**Ensure:** Predicted labels $\hat{y}$, sample-level uncertainty U 1:**Preprocess:** align samples; impute missing values; Z-score normalize each $X^{\left(m\right)}$ (Equations (1) and (2)); select top-2000 features per omics by ANOVA F-test on the training set 2:**Augment:** produce two augmented views per sample using additive Gaussian noise (Equation (3)) and local feature mixing (Equation (5)) 3:**FastMI edges:** for every cross- and within-omics feature pair (x,y), estimate I(x,y) via *k*-NN (Equation (8)); retain pairs with I>τ as candidate dependency edges 4:**Build graph** G=(V,E): sample nodes, omics-specific feature nodes, and three edge types—sample–sample (Equation (10)), sample–feature (Equation (11)), feature–feature (Equation (13)) 5:**for** m=1 **to** *M* **do** 6:    Initialize dual-stream model $F_{m}$ with distinct depth/heads/channels 7:    **while** not converged **and** epoch < max_epochs **do** 8:        **Data stream:** project each $x^{\left(m^{\prime }\right)}$ to $d_{\mathrm{model}}$ and feed into a Transformer encoder (Equations (15)–(18)) to obtain $h_{\mathrm{data}}$ 9:        **Graph stream:** run a 2-layer GAT on G (Equation (20)) to obtain per-sample $h_{\mathrm{graph}}$10:        **Gated fusion:** compute gate α (Equation (22)) and fused embedding $h_{\mathrm{comb}}$ (Equation (23))11:        Backpropagate $L=L_{\mathrm{focal}}+\beta L_{\mathrm{contrast}}+\lambda L_{\mathrm{smooth}}$ (Equation (35))12:    **end while**13:    Store trained model $F_{m}$14:**end for**15:**Uncertainty-aware ensemble:** for each test sample x, compute ${\hat{p}}_{m}\left(x\right)$ (Equation (24)); estimate per-model entropy (Equation (26)); derive adaptive weights $\alpha_{m}\left(x\right)$ (Equation (27)); aggregate ${\hat{p}}_{\mathrm{ens}}\left(x\right)$ (Equation (28)) and predict ${\hat{y}}_{\mathrm{ens}}=\arg\max{\hat{p}}_{\mathrm{ens}}$16:**return** $\hat{y}$, sample-level entropy $U=H\left({\hat{p}}_{\mathrm{ens}}\right)$

### 2.2. Data Preprocessing

We utilize publicly available multi-omics datasets provided by Wang et al. [15], which include four representative cases: ROSMAP (Alzheimer’s disease vs. normal controls), LGG (grade II vs. grade III low-grade gliomas), KIPAN (three kidney cancer subtypes: KICH, KIRC, and KIRP), and BRCA (five breast cancer molecular subtypes). To ensure consistency across different omics layers, we first align samples by removing those with missing or mismatched identifiers, guaranteeing that each row corresponds to the same biological sample across all modalities.

Each dataset consists of mRNA, DNA methylation, and miRNA features, with raw feature counts significantly exceeding sample numbers. To address this high-dimensionality challenge, we apply ANOVA F-test feature selection, retaining the top 2000 most informative features when an omics layer exceeds this threshold. This selection process is performed exclusively on the training set to prevent data leakage. Table 1 summarizes the processed benchmark datasets before training-set feature selection.

To further evaluate FastMI-HGNet on a challenging benchmark without near-perfect class separability, we additionally included the TCGA colon adenocarcinoma (COAD) cohort for early- versus late-stage classification. Batch-effect-normalized multi-omics and clinical data were obtained from the UCSC Xena platform [20]. Only primary tumor samples with matched mRNA expression, DNA methylation, and miRNA expression profiles and an available pathological stage annotation were retained. Following the E2 stage-labeling scheme used in prior TCGA cancer-stage classification studies [21] and the multi-omics benchmark setting of MOMA [12], pathological stages I and II (including their substages) were grouped as early-stage disease (label 0), whereas stages III and IV were grouped as late-stage disease (label 1). Samples annotated as stage 0, carcinoma in situ, stage X, unknown, not reported, or with missing pathological stage were excluded. After patient-level matching and removal of duplicate samples, 276 patients were retained, comprising 155 early-stage and 121 late-stage cases. Unlike the near-separable KIPAN benchmark, early/late-stage COAD classification is recognized as a difficult task and thus provides a more stringent comparison across methods.

To handle missing values resulting from experimental variability and sequencing artifacts, we implement mean imputation. For each omics dataset $X^{\left(m\right)}$ (m=1,2,3 representing mRNA, DNA methylation, and miRNA, respectively) and feature column *j*, missing values are replaced with the mean calculated from available samples:

(1) $$\mu_{j}=\frac{\sum_{i\in S_{j}}x_{i,j}^{\left(m\right)}}{\left|S_{j}\right|}$$

where $S_{j}=\left\{i|x_{i,j}^{\left(m\right)}\neq \mathrm{NaN}\right\}$ represents samples with non-missing values.

Following imputation, we apply Z-score normalization to ensure numerical stability and improve model convergence:

(2) $$x_{i,j}^{\left(\mathrm{scaled},m\right)}=\frac{x_{i,j}^{\left(m\right)}-{\bar{x}}_{j}^{\left(m\right)}}{\sigma_{j}^{\left(m\right)}}$$

where ${\bar{x}}_{j}^{\left(m\right)}$ and $\sigma_{j}^{\left(m\right)}$ denote the mean and standard deviation of feature *j* in omics layer *m*. Importantly, both imputation parameters and normalization statistics are calculated solely from the training data and subsequently applied to validation and test sets, maintaining methodological integrity and preventing information leakage.

### 2.3. Data Augmentation

To enhance model robustness against noise and distributional variations, we implement a multi-view data augmentation strategy after feature selection and normalization. For each omics dataset (mRNA, miRNA, and DNA methylation), we generate perturbed versions of the feature matrix $X^{\left(m\right)}$ while preserving biological integrity.

We employ four augmentation techniques:

1. **Additive Gaussian noise** simulates sequencing errors by adding small perturbations from a normal distribution $N(0,\sigma^{2})$ with σ=0.005, as defined in Equation (3):
  (3) $$x_{i,j}^{\left(\mathrm{noisy},m\right)}=x_{i,j}^{\left(m\right)}+\epsilon ,\;\epsilon ∼N\left(0,\sigma^{2}\right)$$
2. **Random masking** simulates missing values by replacing selected positions with zeros, maintaining a 95% retention rate, as described in Equation (4):
  (4) $$x_{i,j}^{\left(\mathrm{masked},m\right)}=\left\{\begin{array}{cc} x_{i,j}^{\left(m\right)}, & \mathrm{if}\;r_{i,j}>p_{\mathrm{mask}}\\ 0, & \mathrm{otherwise} \end{array}\right.$$
  where $r_{i,j}$ is a uniform random variable in [0,1], and $p_{\mathrm{mask}}$ represents the masking probability.
3. **Local feature mixing** introduces structured perturbations by randomly shuffling sample indices, as formulated in Equation (5):
  (5) $$x_{i,j}^{\left(\mathrm{mixed},m\right)}=x_{\pi (i),j}^{\left(m\right)}$$
  where π(i) represents a random permutation of sample indices.
4. **Random scaling** simulates batch effects by multiplying each feature with a random coefficient, as shown in Equation (6):
  (6) $$x_{i,j}^{\left(\mathrm{scaled},m\right)}=x_{i,j}^{\left(m\right)}\cdot \lambda ,\;\lambda ∼U\left(0.9,1.1\right)$$

To balance computational efficiency with augmentation benefits, we retain only two augmented versions—Gaussian noise (Equation (3)) and local feature mixing (Equation (5))—alongside the original dataset. These perturbations are applied independently to each omics layer and combined with the original training set, effectively increasing sample diversity while maintaining label consistency.

During training, models are trained on this augmented dataset, while validation and test sets remain unaltered to ensure objective performance evaluation. We further leverage the multi-view nature of our augmentation by incorporating contrastive learning in the objective function, as detailed in Equation (7):

(7) $$L_{\mathrm{contrastive}}=\sum_{(i,j)\in P}\left(1-\cos(h_{i},h_{j})\right)+\sum_{(i,k)\in N}\max\left(0,\cos\left(h_{i},h_{k}\right)-m\right)$$

where P and N denote sets of positive and negative sample pairs (augmented versions of the same or different samples), $h_{i}$ represents the feature embedding of sample *i*, and *m* is a margin parameter controlling separation between negative pairs.

This integrated approach enhances model robustness against noise and improves generalization across heterogeneous distributions while effectively capturing multi-omics interactions.

### 2.4. FastMI-Based Statistical Dependency Inference for Edge Priors

To uncover strong nonlinear statistical associations between omics features, we implement a fast mutual information (FastMI) method based on *k*-nearest neighbor estimation, following the estimator of Kraskov, Stögbauer, and Grassberger (KSG) [22]. Mutual information quantifies statistical dependence between feature vectors and is used here to derive dependency edge priors for heterogeneous graph construction. Unlike traditional linear correlation approaches, FastMI captures nonlinear and non-Gaussian dependency patterns. For any two feature vectors $x,y\in R^{N}$ from either the same or different omics layers, we estimate their mutual information using nearest neighbor search in the joint space x⊕y, as defined in Equation (8):

(8) $$I\left(x,y\right)=\psi \left(k\right)-\frac{1}{N}\sum_{i=1}^{N}\left[\psi \left(n_{x,i}+1\right)+\psi \left(n_{y,i}+1\right)\right]+\psi \left(N\right)$$

where ψ(·) denotes the digamma function, *k* is the number of nearest neighbors (adjustable based on dataset size), and *N* is the number of samples. For each sample *i*, we first locate its *k*-th nearest neighbor in the joint space x⊕y and denote the corresponding distance by $\varepsilon_{i}$; $n_{x,i}$ and $n_{y,i}$ are then the numbers of samples lying within distance $\varepsilon_{i}$ of sample *i* in the marginal spaces of x and y, respectively. We emphasize that this *k*-nearest-neighbor (KSG) estimator [22] does not rely on fixed-width or adaptive binning as used by earlier histogram-based mutual-information estimators.

We use a FastMI threshold of τ=0.3 in the main experiments. Its robustness was subsequently examined over τ∈{0.1,0.2,0.3,0.4,0.5}, as described in the Hyperparameter Sensitivity Analysis subsection. Feature pairs exceeding this threshold are incorporated into the heterogeneous graph as FastMI-derived dependency edge priors. When prior biological knowledge establishes directionality, the corresponding edge can be represented as directed; otherwise, relationships are represented as bidirectional or undirected dependency edges.

In our multi-omics framework, we compute FastMI for all possible feature pairs across mRNA, miRNA, and DNA methylation layers, capturing both intra-omics relationships and cross-omics associations. All significant pairs (those with I(x,y)>τ as calculated in Equation (8)) are incorporated into the heterogeneous graph, enabling the subsequent graph neural network to leverage these high-order interactions.

The FastMI approach offers two key advantages over linear correlation methods. First, it provides a more comprehensive representation of statistical dependencies, particularly for non-linear or segmented distributions. Second, by integrating these FastMI-derived dependency edges into the heterogeneous graph, the model incorporates biologically informative cross-omics dependency patterns that can support both classification performance and downstream biological interpretation.

### 2.5. Heterogeneous Graph Construction

After feature selection on our training dataset of *N* samples, we denote the dimensions of mRNA, miRNA, and DNA methylation layers as $d_{1}$, $d_{2}$, and $d_{3}$, respectively. Our heterogeneous graph contains two main categories of nodes:

(1) Sample nodes: $V_{\mathrm{sample}}=\left\{v_{i}^{\left(\mathrm{sample}\right)}|i=1,2,\ldots ,N\right\}$, each representing a biological sample with associated label information.
(2) Omics feature nodes, comprising:
  - mRNA: $V_{\mathrm{mRNA}}=\left\{v_{j}^{\left(\mathrm{mRNA}\right)}|j=1,2,\ldots ,d_{1}\right\}$
  - miRNA: $V_{\mathrm{miRNA}}=\left\{v_{k}^{\left(\mathrm{miRNA}\right)}|k=1,2,\ldots ,d_{2}\right\}$
  - DNA methylation: $V_{\mathrm{meth}}=\left\{v_{\ell }^{\left(\mathrm{meth}\right)}|\ell =1,2,\ldots ,d_{3}\right\}$

Each feature node type receives a distinct label to differentiate it during heterogeneous graph convolution operations.

To model the complex relationships within multi-omics data, we define three types of edges:

**Sample–sample edges**: To enhance high-order neighborhood information, we compute correlation scores between samples. For samples *i* and $i^{\prime }$ with feature vectors $x_{i}$ and $x_{i^{\prime }}$, we calculate their correlation as shown in Equation (9):

(9) $$s\left(i,i^{\prime }\right)=\mathrm{corr}\left(x_{i},x_{i^{\prime }}\right)$$

When $s(i,i^{\prime })$ exceeds the threshold $\tau_{\mathrm{sample}}=0.7$ used in the main experiments, we add an edge between the two sample nodes as defined in Equation (10). Sensitivity to this threshold is evaluated in the Hyperparameter Sensitivity Analysis subsection.

(10) $$e=(v_{i}^{\left(\mathrm{sample}\right)}\to v_{i^{\prime }}^{\left(\mathrm{sample}\right)})$$

**Sample–feature edges**: These connect samples to their strongly deviating (over- or under-regulated) features. Because each feature is Z-score normalized (Equation (2)), the criterion $\left|x_{i,j}^{\left(m\right)}\right|>\tau_{\mathrm{feat}}$ is a two-sided threshold on the standardized value and therefore captures both strongly over- and under-expressed features rather than high expression alone. For sample *i* and feature *j* in omics layer *m*, if $\left|x_{i,j}^{\left(m\right)}\right|>\tau_{\mathrm{feat}}$—with $\tau_{\mathrm{feat}}=2.0$ in standard-deviation units in the main experiments (|z|>2.0, i.e., approximately the most extreme 4.6% of values under a standard normal distribution)—we create an edge as specified in Equation (11). Sensitivity to this threshold is evaluated in the Hyperparameter Sensitivity Analysis subsection.

(11) $$e=\left(v_{i}^{\left(\mathrm{sample}\right)},v_{j}^{\left(m\right)},\mathrm{type}=“\mathrm{assoc}”,w=\left|x_{i,j}^{\left(m\right)}\right|\right)$$

**Feature–feature edges**: These incorporate strong nonlinear statistical dependencies derived from FastMI as dependency edge priors. For features *j* and $j^{\prime }$ in omics layers *m* and $m^{\prime }$ respectively, we evaluate their mutual information according to Equation (12):

(12) $$I\left(x_{\cdot ,j}^{\left(m\right)},x_{\cdot ,j^{\prime }}^{\left(m^{\prime }\right)}\right)>\tau_{\mathrm{fastMI}}$$

If this criterion is met, we add a dependency edge prior as defined in Equation (13):

(13) $$e=\left(v_{j}^{\left(m\right)},v_{j^{\prime }}^{\left(m^{\prime }\right)},\mathrm{type}=“\mathrm{dependency}”,w=I\left(x_{\cdot ,j}^{\left(m\right)},x_{\cdot ,j^{\prime }}^{\left(m^{\prime }\right)}\right)\right)$$

The complete edge set E integrates these three relationship types as described in Equations (10), (11) and (13), forming a heterogeneous multi-modal graph G=(V,E). This structure explicitly encodes sample–feature interactions while preserving multi-scale biological relationships, providing an expressive framework for downstream graph neural network models.

### 2.6. Dual-Stream Fusion Module

In this section, we integrate multi-omics vector-based features with a heterogeneous multi-omics graph to construct a dual-stream framework, consisting of a data stream and a graph stream. These two streams are fused at the output stage using a gated attention mechanism to generate a unified sample representation for downstream classification tasks.

#### 2.6.1. Data Stream

For each sample, the feature vectors from mRNA, miRNA, and DNA methylation omics layers are represented as shown in Equation (14):

(14) $$x_{\mathrm{mRNA}}\in R^{d_{1}},\;x_{\mathrm{miRNA}}\in R^{d_{2}},\;x_{\mathrm{meth}}\in R^{d_{3}}$$

To align the dimensionality across omics data, we apply a linear transformation as defined in Equation (15):

(15) $${\tilde{x}}_{\mathrm{mRNA}}=W_{m}x_{\mathrm{mRNA}},\;{\tilde{x}}_{\mathrm{miRNA}}=W_{r}x_{\mathrm{miRNA}},\;{\tilde{x}}_{\mathrm{meth}}=W_{t}x_{\mathrm{meth}}$$

where $W_{m}$, $W_{r}$, and $W_{t}$ are learnable matrices that project the features into a unified space of dimension $d_{\mathrm{model}}$. The transformed feature vectors are concatenated into a matrix $X\in R^{3\times d_{\mathrm{model}}}$ and passed through a multi-head self-attention module as shown in Equation (16):

(16) $$\mathrm{Attn}\left(Q,K,V\right)=\mathrm{softmax}\left(\frac{QK^{⊤}}{\sqrt{d_{\mathrm{head}}}}\right)V$$

where Q, K, and V are obtained from the linear projections of X, and $d_{\mathrm{head}}=d_{\mathrm{model}}/\mathrm{heads}$. The outputs from all attention heads are concatenated and projected to form $X_{\mathrm{attn}}$.

Following the attention module, a standard Transformer residual block and feedforward network (FFN) further refine feature interactions. Given a position vector z, the residual connection is computed as defined in Equation (17):

(17) $$z^{\prime }=\mathrm{LayerNorm}\left(z+\mathrm{Dropout}\left(\mathrm{MultiHeadAttn}(z)\right)\right)$$

The feedforward network processing is shown in Equation (18):

(18) $$z^{\prime \prime }=\mathrm{LayerNorm}\left(z^{\prime }+\mathrm{Dropout}\left(\mathrm{GELU}\left(W_{2}\;\mathrm{GELU}\left(W_{1}z^{\prime }+b_{1}\right)+b_{2}\right)\right)\right)$$

where $W_{1}$, $W_{2}$ and $b_{1}$, $b_{2}$ are learnable parameters, and GELU is the activation function. Dropout and LayerNorm are applied to mitigate overfitting and accelerate convergence.

The final output $Z^{\prime \prime }\in R^{3\times d_{\mathrm{model}}}$ is concatenated and linearly projected into a single vector as represented in Equation (19):

(19) $$h_{\mathrm{data}}\in R^{d_{d}}$$

which serves as the global representation of the data stream.

#### 2.6.2. Graph Stream

The heterogeneous multi-omics graph G=(V,E) consists of sample nodes and feature nodes (mRNA, miRNA, and DNA methylation), along with various edge types, including FastMI-derived dependency edge priors. To capture high-order dependencies in the graph, we employ a graph attention network (GAT), which updates node representations in each layer as defined in Equation (20):

(20) $$h_{v}^{\left(l+1\right)}=\sigma \left(\sum_{u\in N(v)}\alpha_{vu}^{\left(l\right)}W^{\left(l\right)}h_{u}^{\left(l\right)}\right)$$

where: $\alpha_{vu}^{\left(l\right)}$ is the attention weight assigned to neighbor node *u* when aggregating its information into node *v*, $W^{\left(l\right)}$ is a learnable parameter matrix, and σ(·) represents an activation function such as ReLU or GELU.

We stack two GAT layers to allow each sample node to receive second-order neighborhood information from feature nodes. The final graph representation for each sample node $v_{i}^{\left(\mathrm{sample}\right)}$ is extracted as shown in Equation (21):

(21) $$h_{v_{i}}^{\left(\mathrm{graph}\right)}\in R^{d_{g}}$$

#### 2.6.3. Gated Attention Fusion

For each sample *i*, we obtain both the data stream representation $h_{\mathrm{data},i}\in R^{d_{d}}$ and the graph stream representation $h_{\mathrm{graph},i}\in R^{d_{g}}$. To adaptively integrate both representations, we employ a gated attention mechanism, which learns a weighting factor $\alpha_{i}\in \left[0,1\right]$ as defined in Equation (22):

(22) $$\alpha_{i}=\sigma \left(W_{g}\left[h_{\mathrm{data},i}\;∥\;h_{\mathrm{graph},i}\right]+b_{g}\right)$$

where: $W_{g}$ and $b_{g}$ are learnable parameters, σ(·) is the sigmoid activation function, and ‖ denotes vector concatenation.

The final fused representation is computed according to Equation (23):

(23) $$h_{\mathrm{comb},i}=\alpha_{i}⊙h_{\mathrm{data},i}+\left(1-\alpha_{i}\right)⊙h_{\mathrm{graph},i}$$

where ⊙ represents element-wise multiplication. This mechanism allows the model to dynamically adjust the weighting between vector-based and graph-based representations based on sample characteristics.

The final fused vector $h_{\mathrm{comb},i}\in R^{\max(d_{d},d_{g})}$ is then passed to the classification head for disease classification, including tumor subtyping when applicable.

### 2.7. Uncertainty-Aware Ensemble Prediction

In this section, we propose an uncertainty-aware ensemble strategy that combines multiple dual-stream fusion models to enhance prediction robustness and reliability. This approach addresses variations in parameter initialization, network architecture, and inherent data noise while providing confidence estimates for model interpretation and future validation.

We train an ensemble of *M* dual-stream models, denoted as $F_{1},F_{2},\ldots ,F_{M}$, each with distinct configurations (varying network depth, attention head count, or graph convolution channels) to capture diverse perspectives on multi-omics data patterns. For an input sample x comprising omics features $x_{\mathrm{mRNA}}$, $x_{\mathrm{miRNA}}$, $x_{\mathrm{meth}}$ and corresponding graph information, each model $F_{m}$ generates a predictive distribution as shown in Equation (24):

(24) $${\hat{p}}_{m}\left(x\right)=\mathrm{softmax}\left(F_{m}\left(x\right)\right)$$

where $F_{m}\left(x\right)$ represents the output logits before softmax transformation, with dimensionality equal to the number of classes *C*.

Traditional ensemble methods typically combine predictions through weighted voting or simple averaging, as expressed in Equation (25):

(25) $${\hat{p}}_{\mathrm{ens}}\left(x\right)=\sum_{m=1}^{M}\alpha_{m}\;{\hat{p}}_{m}\left(x\right)$$

where weights $\alpha_{m}$ satisfy $\alpha_{m}\geq 0$ and $\sum_{m}\alpha_{m}=1$.

However, in complex multi-omics applications, models often exhibit varying confidence levels across different samples. To address this challenge, we introduce a sample-specific adaptive weighting mechanism based on model uncertainty estimation.

We quantify prediction uncertainty using the information-theoretic entropy measure defined in Equation (26):

(26) $$\mathrm{Uncert}\left({\hat{p}}_{m}\left(x\right)\right)=-\sum_{c=1}^{C}{\hat{p}}_{m}^{\left(c\right)}\left(x\right)\ln\left({\hat{p}}_{m}^{\left(c\right)}\left(x\right)\right)$$

where ${\hat{p}}_{m}^{\left(c\right)}\left(x\right)$ represents the predicted probability of class *c* from model *m*. Higher entropy values indicate greater uncertainty, suggesting that the model lacks a clear preference among possible classes.

To prioritize models with higher confidence in the ensemble, we transform uncertainty into an adaptive weighting factor using Equation (27):

(27) $$\alpha_{m}\left(x\right)=\frac{\exp\left(-\mathrm{Uncert}\left({\hat{p}}_{m}\left(x\right)\right)\right)}{\sum_{m^{\prime }=1}^{M}\exp\left(-\mathrm{Uncert}\left({\hat{p}}_{m^{\prime }}\left(x\right)\right)\right)}$$

This formulation ensures that models with lower entropy (higher confidence) receive greater weight for each specific sample x, while less confident models are downweighted accordingly. The final uncertainty-weighted ensemble prediction is computed as shown in Equation (28):

(28) $${\hat{p}}_{\mathrm{ens}}\left(x\right)=\sum_{m=1}^{M}\alpha_{m}\left(x\right)\;{\hat{p}}_{m}\left(x\right)$$

The predicted class label is then determined according to Equation (29):

(29) $${\hat{y}}_{\mathrm{ens}}=\arg\max{\hat{p}}_{\mathrm{ens}}\left(x\right)$$

Beyond improving classification accuracy, our uncertainty-aware ensemble approach provides confidence metrics for model interpretation. The entropy of the final ensemble distribution ${\hat{p}}_{\mathrm{ens}}\left(x\right)$ serves as a global uncertainty measure for each prediction. Samples with consistently high uncertainty across all models may represent inherently ambiguous cases or outliers that warrant additional investigation. This uncertainty quantification can help identify ambiguous or low-confidence predictions that warrant further investigation in future validation studies.

### 2.8. Training Objective and Implementation Details

In this section, we present a comprehensive training framework designed to optimize classification performance while enhancing model robustness and generalization capabilities. Our approach employs a composite loss function that integrates classification loss with contrastive learning and regularization techniques specifically tailored for multi-omics data analysis.

#### 2.8.1. Primary Task Loss

To address class imbalance and hard-to-classify samples commonly encountered in multi-omics disease classification datasets, we implement Focal Loss as our primary classification objective. As shown in Equation (30), for a sample *i* with ground-truth label $y_{i}$ and model output logits $z_{i}\in R^{C}$ (where *C* is the number of classes), the corresponding softmax probability is

(30) $${\hat{p}}_{i}^{\left(c\right)}=\frac{\exp(z_{i}^{\left(c\right)})}{\sum_{c^{\prime }=1}^{C}\exp\left(z_{i}^{\left(c^{\prime }\right)}\right)}$$

The Focal Loss function, defined in Equation (31), dynamically adjusts the learning focus toward difficult examples:

(31) $$L_{\mathrm{focal}}\left(z_{i},y_{i}\right)=-\alpha_{y_{i}}\;{\left(1-{\hat{p}}_{i}^{\left(y_{i}\right)}\right)}^{\gamma }\;\ln\left({\hat{p}}_{i}^{\left(y_{i}\right)}\right)$$

where γ is the focusing parameter that modulates the rate at which easy examples are downweighted (typically set to 2), and $\alpha_{y_{i}}$ represents the class-specific weight that counterbalances class frequency disparities. For datasets with minimal class imbalance, we can substitute Focal Loss with standard cross-entropy loss.

#### 2.8.2. Contrastive Learning Loss

To leverage the multi-view nature of omics data, we incorporate contrastive learning principles that treat different augmentations of the same sample as positive pairs while considering distinct samples as negative pairs. This approach encourages the model to develop representations that are invariant to data perturbations while maintaining discriminative power between different samples.

The contrastive loss function is formulated as shown in Equation (32):

(32) $$L_{\mathrm{contrast}}=\frac{1}{N_{\mathrm{pos}}}\sum_{\mathrm{pos}}\left(1-\cos(h_{i},h_{j})\right)+\frac{1}{N_{\mathrm{neg}}}\sum_{\mathrm{neg}}\max\left(0,\cos(h_{i},h_{j})-m\right)$$

where cos(·,·) represents cosine similarity between feature vectors, *m* is a margin parameter (typically 0.5), and $N_{\mathrm{pos}}$ and $N_{\mathrm{neg}}$ denote the numbers of positive and negative pairs, respectively. This loss function explicitly enhances the model’s ability to maintain consistent representations for the same sample under various perturbations while increasing the separation between different samples in the embedding space.

#### 2.8.3. Label Smoothing Regularization

To prevent overconfidence and improve generalization, we incorporate label smoothing regularization. Instead of training with one-hot encoded labels that encourage the model to assign probability 1 to the correct class and 0 to all others, label smoothing introduces a small uncertainty factor. The smoothed label distribution $q^{\prime }$ is defined as

(33) $$q_{i}^{\prime (c)}=\left\{\begin{array}{cc} 1-\epsilon +\frac{\epsilon }{C}, & \mathrm{if}\;c=y_{i}\\ \frac{\epsilon }{C}, & \mathrm{otherwise} \end{array}\right.$$

where ϵ∈[0,1] is the smoothing parameter, typically set to 0.1. The corresponding loss function is

(34) $$L_{\mathrm{smooth}}\left(z_{i},y_{i}\right)=-\sum_{c=1}^{C}q_{i}^{\prime (c)}\log\left({\hat{p}}_{i}^{\left(c\right)}\right)$$

This approach prevents the model from becoming overly confident in its predictions on training samples, thereby reducing overfitting and improving robustness to noisy labels.

#### 2.8.4. Final Training Objective

The final objective function integrates the primary classification loss, contrastive loss, and label smoothing regularization as shown in Equation (35):

(35) $$L=L_{\mathrm{focal}}\left(z,y\right)+\beta L_{\mathrm{contrast}}\left(h\right)+\lambda L_{\mathrm{smooth}}\left(z,y\right)$$

where z represents the output logits from the classification layer, h denotes the learned sample embeddings from the dual-stream fusion representation $h_{\mathrm{comb}}$, β controls the contribution of contrastive learning, and λ determines the strength of label smoothing regularization.

To efficiently search for suitable values of β and λ (as well as other critical hyperparameters, such as the Focal Loss gamma), we employ a simplified Bayesian optimization procedure. Specifically, we use the Tree-structured Parzen Estimator to model the objective function over the hyperparameter space. We initialize prior distributions for each hyperparameter (e.g., uniform or log-uniform within a reasonable range), then iteratively update these distributions based on the observed performance in a *k*-fold cross-validation setting. This approach adaptively balances exploration and exploitation, guiding the search toward more promising hyperparameter configurations. After a predefined number of iterations, we select the combination of β, λ, and other hyperparameters that yields the highest average cross-validation accuracy (or a composite metric if desired) as our final setting.

Through this process, we effectively balance the primary classification objective with representation learning and regularization constraints, supporting stable performance across multi-omics disease classification tasks. In our experiments, we found that values of β and λ in the range [0.01, 0.1] and [0.05, 0.2], respectively, often perform well across multiple datasets.

#### 2.8.5. Implementation Details

Our framework was implemented using PyTorch version 2.6.0, and all experiments were conducted on NVIDIA A100 GPUs. For model training, the entire dataset was first split into a training set (80%) and an independent test set (20%) using stratified sampling based on the class labels to ensure proportional representation. We employed the AdamW optimizer with an initial learning rate of $3\times 10^{-4}$ and a weight decay of $1\times 10^{-5}$. The learning rate was dynamically adjusted using a cosine annealing schedule with warm restarts to facilitate stable convergence. Models were trained for a maximum of 200 epochs with a batch size of 32. To prevent overfitting, we implemented an early stopping criterion with a patience of 20 epochs, monitored on the validation loss. The composite loss function was configured with a Focal Loss focusing parameter γ of 2.0 and class weights set inversely proportional to class frequencies. For the regularization components, the contrastive loss used a temperature τ of 0.07 and a weighting factor β of 0.1, while label smoothing was applied with a coefficient λ of 0.1. Final model selection and hyperparameter tuning were performed using a 5-fold cross-validation strategy on the training set, and the model’s ultimate performance was evaluated on the held-out independent test set.

Gene Ontology (GO) Biological Process enrichment analysis was performed using the clusterProfiler R package (v4.4.4) with org.Hs.eg.db as the gene universe annotation database. Gene symbols were converted to Entrez IDs using bitr. Enrichment *p*-values were computed using Fisher’s exact test, and GO terms with nominal p<0.05 were considered enriched. Multiple-testing-adjusted *p*-values were reported in the GO visualization for reference.

### 2.9. Computational Complexity and Scalability

After ANOVA feature selection, each dataset comprises *P* feature nodes (2000 mRNA + 2000 DNA methylation + all miRNA features; see Table 1) together with the training-set sample nodes. FastMI evaluates all P2 intra- and cross-omics feature pairs, corresponding to approximately 0.9–$1.3\times 10^{7}$ pairs per dataset (about $5.2\times 10^{7}$ pairs across the five benchmarks); after thresholding at τ=0.3, only the strongest dependency pairs are retained, yielding a sparse heterogeneous graph.

Let *N* denote the number of training samples and *P* the total number of features. FastMI estimates the mutual information of each feature pair using a KD-tree-based *k*-nearest-neighbor search with per-pair complexity O(NlogN), so the overall edge-construction time complexity is $O(P^{2}\;N\log N)$. In terms of space, the feature matrix requires O(NP), whereas the dependency edges are stored sparsely as O(|E|) after thresholding ($\left|E\right|\ll P^{2}$). Each graph-attention layer scales linearly with the number of edges, i.e., O(|E|d).

Table 2 reports the measured cost on a single NVIDIA A100 GPU. FastMI dependency-edge construction took between 3.13 and 19.63 min across datasets, heterogeneous graph construction completed within 2.3 s, and the peak GPU memory usage was only 0.20–0.34 GiB. Because this step is a one-time preprocessing stage performed before training and is not repeated at each training iteration, its cost is small relative to the overall pipeline.

The pairwise computation scales as $O(P^{2})$ with the number of features. When a larger feature set is biologically necessary (e.g., retaining all ∼20,000 genes), the cost can be controlled by (i) pre-filtering with inexpensive variance- or correlation-based screening before computing mutual information, (ii) accelerating the *k*-nearest-neighbor search with approximate nearest-neighbor methods, and (iii) using multi-core/GPU parallelism together with block-wise computation to bound peak memory. The low peak-memory footprint (below 0.35 GiB) indicates that memory is not a bottleneck; the dominant cost is computation time, which is amenable to parallelization.

## 3. Results

### 3.1. Performance Comparison with Existing Methods

To evaluate the effectiveness of the proposed method, we conduct experiments on five publicly available multi-omics datasets: ROSMAP, LGG, KIPAN, BRCA, and COAD. We systematically compare our approach with 16 existing multi-omics integration methods, including early fusion strategies (e.g., SVM, KNN, Lasso, RF, XGBoost) and recent deep learning-based fusion models (e.g., CF, TMC, GMU, Dynamic, MLCLNet, CrossTrans).

For binary classification tasks (ROSMAP, LGG, and COAD), we use accuracy (ACC), F1-score (F1), and area under the curve (AUC) as evaluation metrics. For multi-class classification tasks (BRCA and KIPAN), we additionally report weighted F1-score (F1_weighted_) and macro-average F1-score (F1_macro_). The experimental results for each dataset are summarized below.

Table 3 reports the classification results on the two original binary-classification benchmarks (ROSMAP and LGG). For the ROSMAP dataset, which differentiates Alzheimer’s disease (AD) from normal controls (NC), traditional early fusion methods such as SVM [23], KNN [24], and Lasso [25] exhibit relatively lower performance, particularly in terms of AUC, indicating their limitations in capturing cross-omics interactions.

Recent multi-omics fusion approaches such as CF [26], TMC [27], and Dynamic [18] significantly enhance classification performance, achieving AUC values above 0.88. CrossTrans [28] achieves an AUC of 0.897, while MLCLNet [19] attains the highest F1-score of 0.852. Dynamic [18] achieves the highest AUC of 0.912.

Compared with these methods, FastMI-HGNet achieves an ACC of 0.885, F1-score of 0.879, and AUC of 0.911. Although Dynamic achieves a slightly higher AUC (0.912), FastMI-HGNet shows higher ACC (0.885 vs. 0.842) and F1-score (0.879 vs. 0.846), indicating competitive performance on the ROSMAP benchmark.

For the LGG dataset, which differentiates between Grade II and Grade III gliomas, traditional early fusion baselines, such as SVM [23], Lasso [25], and RF [29], achieve accuracy and AUC scores in the range of 0.74–0.76, indicating limited effectiveness in capturing cross-omics relationships. Notably, BSPLSDA [30] underperforms on this dataset, with an AUC of 0.730.

In contrast, advanced multi-omics integration methods, including CF [26] and TMC [27], improve the classification performance, achieving an ACC of approximately 0.81 and an AUC exceeding 0.87. More recent deep learning-based fusion approaches, such as Dynamic [18] and MLCLNet [19], further increase the AUC to 0.885–0.886.

Our proposed framework achieves an AUC of 0.885, with an ACC of 0.836 and an F1-score of 0.838, demonstrating a balanced and stable classification performance. Although Dynamic achieves a similar AUC (0.885), our model surpasses it in ACC (0.836 vs. 0.833) and F1-score (0.838 vs. 0.837). This suggests that our approach provides a more comprehensive multi-omics feature representation, ensuring both classification accuracy and robustness.

Table 4 reports the classification results on the two multi-class benchmarks (BRCA and KIPAN). For the BRCA dataset, which involves distinguishing between five molecular subtypes (Basal-like, HER2-enriched, Luminal A, Luminal B, and Normal-like), the task is significantly more challenging than binary classification due to the increased class complexity and potential class imbalance.

As shown in the results, traditional single-omics methods such as SVM [23], KNN [24], and Lasso [25] achieve accuracy around 0.73, while their macro-average F1-score (F1_macro_) remains in the range of 0.64–0.68, indicating limited ability to differentiate between subtypes. Notably, BPLSDA [30] and BSPLSDA [30] perform particularly poorly on this complex task, with F1_macro_ scores of only 0.369 and 0.351, respectively. These results suggest that such methods struggle to effectively distinguish between multiple breast cancer subtypes.

In contrast, multi-modal fusion methods show clear performance gains. CF [26] and TMC [27] achieve ACC scores of 0.815 and 0.842, respectively, with F1_macro_ improving to 0.771 and 0.806, supporting the value of multi-omics integration for complex classification tasks. More recent models, including Dynamic [18], MLCLNet [19], and CrossTrans [28], further increase ACC to the 0.86–0.88 range and F1_macro_ to 0.83–0.85, showing the benefit of advanced fusion architectures.

**Table 3:** Classification results on the binary-classification benchmarks ROSMAP and LGG (mean ± standard deviation).

Method	ROSMAP			LGG		
	ACC	F1	AUC	ACC	F1	AUC
SVM [23]	0.770 ± 0.024	0.778 ± 0.016	0.770 ± 0.026	0.754 ± 0.046	0.757 ± 0.050	0.754 ± 0.046
KNN [24]	0.657 ± 0.036	0.671 ± 0.045	0.709 ± 0.045	0.729 ± 0.034	0.738 ± 0.033	0.799 ± 0.038
Lasso [25]	0.694 ± 0.037	0.730 ± 0.033	0.770 ± 0.035	0.761 ± 0.018	0.767 ± 0.022	0.823 ± 0.027
RF [29]	0.726 ± 0.029	0.734 ± 0.021	0.811 ± 0.019	0.748 ± 0.012	0.742 ± 0.010	0.823 ± 0.010
XGBoost [31]	0.760 ± 0.046	0.772 ± 0.045	0.837 ± 0.030	0.756 ± 0.040	0.767 ± 0.032	0.840 ± 0.023
FCNN [32]	0.755 ± 0.021	0.764 ± 0.021	0.827 ± 0.025	0.737 ± 0.023	0.748 ± 0.024	0.810 ± 0.037
BPLSDA [30]	0.742 ± 0.024	0.755 ± 0.023	0.830 ± 0.025	0.759 ± 0.025	0.738 ± 0.031	0.825 ± 0.023
BSPLSDA [30]	0.753 ± 0.033	0.764 ± 0.035	0.838 ± 0.021	0.685 ± 0.025	0.662 ± 0.030	0.730 ± 0.026
GRidge [33]	0.760 ± 0.034	0.769 ± 0.029	0.841 ± 0.023	0.746 ± 0.038	0.756 ± 0.036	0.826 ± 0.044
CF [26]	0.784 ± 0.011	0.788 ± 0.005	0.880 ± 0.005	0.811 ± 0.012	0.822 ± 0.004	0.881 ± 0.004
TMC [27]	0.825 ± 0.009	0.823 ± 0.006	0.885 ± 0.006	0.819 ± 0.008	0.815 ± 0.004	0.871 ± 0.004
GMU [34]	0.776 ± 0.025	0.784 ± 0.016	0.869 ± 0.016	0.803 ± 0.015	0.808 ± 0.012	0.886 ± 0.012
MOGONET [15]	0.815 ± 0.023	0.821 ± 0.022	0.874 ± 0.012	0.816 ± 0.016	0.814 ± 0.027	0.840 ± 0.027
Dynamic [18]	0.842 ± 0.013	0.846 ± 0.007	0.912 ± 0.007	0.833 ± 0.010	0.837 ± 0.004	0.885 ± 0.004
MLCLNet [19]	0.844 ± 0.015	0.852 ± 0.015	0.893 ± 0.011	0.835 ± 0.014	0.840 ± 0.013	0.886 ± 0.012
CrossTrans [28]	0.865 ± 0.013	0.863 ± 0.008	0.897 ± 0.012	0.826 ± 0.017	0.824 ± 0.013	0.881 ± 0.007
FastMI-HGNet	0.885 ± 0.003	0.879 ± 0.009	0.911 ± 0.011	0.836 ± 0.012	0.838 ± 0.008	0.885 ± 0.006

FastMI-HGNet achieves the best overall performance on the BRCA dataset, with an ACC of 0.881, F1_weighted_ of 0.885, and F1_macro_ of 0.859. Compared with the second-ranked Dynamic model, FastMI-HGNet improves F1_macro_ by 0.014, suggesting improved balance in this imbalanced five-class molecular subtyping task. This performance can be attributed in part to the FastMI-based statistical dependency inference mechanism, which identifies informative cross-omics features from different omics layers that are relevant to molecular subtyping.

For the KIPAN dataset, which involves distinguishing between three kidney cancer subtypes (KIRC, KIRP, and KICH), all methods achieve high classification accuracy, indicating that distinct molecular features differentiate the KIPAN subtypes.

Traditional single-omics methods already perform well on this dataset. For example, SVM [23] and XGBoost [31] achieve ACC values of 0.995 and 0.993, respectively, with F1_macro_ scores of 0.994 and 0.989. However, BPLSDA [30] and BSPLSDA [30] perform relatively poorly, with ACC values of 0.933 and 0.919, and F1_macro_ scores of 0.919 and 0.895, respectively. These results indicate that these methods face limitations when handling multi-omics data.

Multi-modal fusion methods further enhance classification performance. Notably, MOGONET [15], Dynamic [18], MLCLNet [19], and CrossTrans [28] all achieve nearly perfect classification performance, with ACC, F1_weighted_, and F1_macro_ reaching 0.999. This suggests that on datasets where subtypes exhibit clear molecular differences, advanced multi-modal fusion methods can fully leverage complementary omics information to achieve near-perfect classification.

FastMI-HGNet also achieves top-tier performance on the KIPAN dataset, with an ACC of 0.999, F1_weighted_ of 0.999, and F1_macro_ of 0.999, matching the best-performing methods. Because numerical differences between methods are minimal on this highly separable dataset, KIPAN is best viewed as a ceiling-level benchmark; ROSMAP, LGG, BRCA, and COAD provide more informative settings for assessing model generalization. Notably, even in this relatively straightforward classification task, FastMI-HGNet maintains competitive performance with minimal standard deviation, indicating stable behavior under favorable benchmark conditions.

We also examined whether the near-perfect KIPAN performance could be explained by a single discriminative feature. Logistic regression using the single most discriminative mRNA feature (selected by ANOVA F-test, F-statistic = 872) achieved 61.2% test-set accuracy, indicating that the task is not solved by one feature alone. The near-perfect results in Table 4 are therefore more consistent with broad multi-omics separation among the three renal cancer subtypes (KICH, KIRC, KIRP), in line with the original benchmark study [15]. We accordingly treat KIPAN as a multi-class proof-of-concept benchmark and emphasize ROSMAP, LGG, BRCA, and COAD when interpreting discriminative performance.

**Table 4:** Classification results on the multi-class benchmarks BRCA and KIPAN (mean ± standard deviation).

Method	BRCA			KIPAN		
	ACC	F1_weighted_	F1_macro_	ACC	F1_weighted_	F1_macro_
SVM [23]	0.729 ± 0.018	0.702 ± 0.015	0.640 ± 0.017	0.995 ± 0.003	0.995 ± 0.003	0.994 ± 0.004
KNN [24]	0.742 ± 0.024	0.730 ± 0.023	0.682 ± 0.025	0.967 ± 0.011	0.967 ± 0.011	0.960 ± 0.014
Lasso [25]	0.732 ± 0.012	0.698 ± 0.015	0.642 ± 0.026	0.974 ± 0.002	0.974 ± 0.002	0.972 ± 0.004
RF [29]	0.754 ± 0.009	0.733 ± 0.010	0.649 ± 0.013	0.981 ± 0.006	0.981 ± 0.006	0.975 ± 0.011
XGBoost [31]	0.781 ± 0.008	0.764 ± 0.010	0.701 ± 0.017	0.993 ± 0.008	0.993 ± 0.008	0.989 ± 0.014
FCNN [32]	0.754 ± 0.028	0.740 ± 0.034	0.668 ± 0.047	0.991 ± 0.005	0.991 ± 0.005	0.991 ± 0.005
BPLSDA [30]	0.642 ± 0.009	0.534 ± 0.014	0.369 ± 0.017	0.933 ± 0.013	0.933 ± 0.013	0.919 ± 0.021
BSPLSDA [30]	0.639 ± 0.008	0.522 ± 0.016	0.351 ± 0.022	0.919 ± 0.012	0.918 ± 0.013	0.895 ± 0.014
GRidge [33]	0.745 ± 0.016	0.726 ± 0.019	0.656 ± 0.025	0.994 ± 0.004	0.994 ± 0.004	0.993 ± 0.004
CF [26]	0.815 ± 0.008	0.815 ± 0.009	0.771 ± 0.009	0.992 ± 0.005	0.992 ± 0.005	0.988 ± 0.009
TMC [27]	0.842 ± 0.005	0.844 ± 0.009	0.806 ± 0.009	0.997 ± 0.003	0.997 ± 0.003	0.994 ± 0.005
GMU [34]	0.800 ± 0.039	0.798 ± 0.058	0.746 ± 0.058	0.977 ± 0.016	0.976 ± 0.017	0.958 ± 0.032
MOGONET [15]	0.829 ± 0.018	0.825 ± 0.016	0.774 ± 0.017	0.999 ± 0.002	0.999 ± 0.002	0.999 ± 0.002
Dynamic [18]	0.877 ± 0.003	0.880 ± 0.005	0.845 ± 0.005	0.999 ± 0.002	0.999 ± 0.002	0.999 ± 0.003
MLCLNet [19]	0.864 ± 0.016	0.878 ± 0.015	0.826 ± 0.018	0.999 ± 0.003	0.999 ± 0.002	0.999 ± 0.002
CrossTrans [28]	0.862 ± 0.020	0.869 ± 0.012	0.853 ± 0.008	0.999 ± 0.003	0.999 ± 0.002	0.999 ± 0.002
FastMI-HGNet	0.881 ± 0.009	0.885 ± 0.006	0.859 ± 0.005	0.999 ± 0.002	0.999 ± 0.003	0.999 ± 0.003

To further assess FastMI-HGNet under a genuinely challenging setting, Table 5 presents the classification results on the COAD early- versus late-stage benchmark. Consistent with the recognized difficulty of predicting tumor stage from molecular profiles, all methods scored substantially lower than on the subtype-classification benchmarks, with AUC values ranging from 0.561 to 0.708. The majority-class baseline on this cohort corresponds to an accuracy of 0.562, and several classical baselines (KNN [24], BSPLSDA [30], and Lasso [25]) performed at or near this level, underscoring the difficulty of the task. Deep multi-omics fusion methods again outperformed classical approaches, with Dynamic [18] and CrossTrans [28] reaching AUCs of 0.698 and 0.708, respectively. FastMI-HGNet achieved the highest ACC (0.638) and positive-class F1 (0.655) and the second-highest AUC (0.702, versus 0.708 for CrossTrans), while exhibiting the smallest standard deviation across all three metrics. These results indicate that, on a genuinely challenging multi-omics benchmark without a performance ceiling, FastMI-HGNet retains a competitive and stable advantage over both classical and recent deep-learning baselines.

Overall, FastMI-HGNet shows competitive classification performance across datasets with different levels of complexity. The KIPAN benchmark is highly separable and is therefore best regarded as a ceiling-level proof-of-concept benchmark, whereas ROSMAP, LGG, BRCA, and the challenging COAD staging task provide more informative settings for evaluating model discriminative ability and stability.

### 3.2. Module Removal Ablation Study

To evaluate the contributions of key components in our model, we conduct ablation studies by systematically removing the graph neural network (GNN), Transformer module, and FastMI-derived dependency edge priors. The experiments are performed on the four original multi-omics benchmarks, with results for binary classification tasks (ROSMAP and LGG) presented in Table 6, while results for multi-class classification tasks (BRCA and KIPAN) are shown in Table 7.

As shown in the tables, removing the GNN module leads to a noticeable drop in ACC and F1-score across the four original benchmarks, highlighting the importance of heterogeneous graph convolution in capturing high-order topological interactions and dependency patterns. Similarly, removing the Transformer-based feature fusion module reduces model performance, particularly in complex multi-class classification scenarios such as BRCA, where the attention-based architecture plays a crucial role in integrating multi-omics features.

Additionally, eliminating FastMI-derived dependency edge priors (-FastMI) results in a slight decrease in AUC (or F1_macro_ in multi-class settings), indicating that the dependency priors provide complementary information beyond simple feature correlations. Overall, each core module contributes to the final model performance, and their integration enables robust multi-omics classification.

As observed in the tables, removing the GNN and Transformer modules leads to performance degradation in multi-class classification tasks (BRCA, KIPAN), particularly in F1_macro_, indicating a reduced ability to maintain classification balance across different subtypes. While FastMI shows limited impact on the KIPAN dataset due to its inherently distinct subtypes, it provides noticeable performance gains in more challenging datasets such as ROSMAP, LGG, and BRCA.

Overall, the combination of GNN, Transformer, and FastMI modules helps the model maintain stable classification performance in high-dimensional multi-omics scenarios. To facilitate direct comparison with all evaluated methods, we additionally include a *Best Baseline* row in Table 6 and Table 7, reporting for each dataset the strongest performance achieved by any of the 16 baseline methods evaluated in our main benchmark (ROSMAP and BRCA: Dynamic; LGG: MLCLNet; KIPAN: multiple methods reaching ∼0.999 ceiling). Even after removing key modules, the ablated models remain competitive with the strongest baseline on several metrics, particularly on the more challenging BRCA five-class and ROSMAP/LGG binary tasks.

### 3.3. Hyperparameter Sensitivity Analysis

We further examined the sensitivity of FastMI-HGNet to the three thresholds used in heterogeneous graph construction: the FastMI dependency threshold $\tau_{\mathrm{FastMI}}$ (feature–feature edges), the sample–sample correlation threshold $\tau_{\mathrm{sample}}$, and the sample–feature threshold $\tau_{\mathrm{feat}}$. For each threshold we varied its value while keeping the others fixed at the values used in the main experiments, and report the resulting test-set accuracy in Table 8.

Across all three thresholds, performance follows an inverted-U trend: it degrades when the threshold is too low (an overly dense graph introduces spurious edges and noise) or too high (an overly sparse graph discards useful structure), and peaks near the values used in the main experiments ($\tau_{\mathrm{FastMI}}=0.3$, $\tau_{\mathrm{sample}}=0.7$, and $\tau_{\mathrm{feat}}=\left|z\right|>2.0$). Performance is stable within a broad band around these values (variation within roughly 1% for $\tau_{\mathrm{FastMI}}\in \left[0.2,0.4\right]$, $\tau_{\mathrm{sample}}\in \left[0.5,0.9\right]$, and $\tau_{\mathrm{feat}}\in \left[1.5,2.5\right]$), indicating that the model is robust to the exact threshold choice and that these values achieve the best or near-best trade-off.

Two consistent patterns emerge. First, the sensitivity scales with task difficulty: the near-separable KIPAN benchmark is essentially insensitive to all thresholds (variation ≤0.001), whereas the more challenging COAD staging task is the most sensitive (up to ∼0.013), consistent with harder tasks relying more on the graph-derived structure. Second, among the three thresholds, $\tau_{\mathrm{FastMI}}$ has the largest overall impact, in line with the central role of FastMI-derived dependency edges in the model. Because $\tau_{\mathrm{feat}}$ operates on Z-score-normalized values, it is reported in standard-deviation units together with the corresponding two-sided retention percentiles, confirming that the sample–feature edges capture both strongly over- and under-expressed features rather than high expression alone.

### 3.4. Statistical Significance

To assess the statistical reliability of the reported performance, we computed bootstrap 95% confidence intervals (1000 resamples of the held-out test set) for FastMI-HGNet on the primary metric of each benchmark, and performed paired significance tests against a representative classical baseline (SVM) and a recent high-performing comparator for each dataset (Table 9). AUC comparisons used the DeLong test, and F1_macro_ comparisons used a paired bootstrap test.

FastMI-HGNet significantly outperformed the classical early-fusion baseline on the discriminative benchmarks (ROSMAP, LGG, BRCA, and COAD; p<0.01), whereas its differences from the recent deep-learning comparators were not statistically significant, with overlapping confidence intervals. This indicates that, on these small-sample benchmarks, FastMI-HGNet is statistically comparable to state-of-the-art methods; its advantages lie in consistent performance across metrics and tasks, in stability (the smallest standard deviation), and in interpretability, rather than in a single-metric margin. On the near-separable KIPAN benchmark, all methods are statistically indistinguishable, consistent with its ceiling-level nature.

### 3.5. Controlled Simulation Study for FastMI Validation

To evaluate FastMI’s reliability under controlled conditions with known ground-truth dependencies, we conducted a simulation study on synthetic multi-omics data. Two feature matrices $X,Y\in R^{200\times 60}$ were generated from i.i.d. standard Gaussian distributions, with K=8 randomly chosen feature pairs (i,j) planted with non-linear dependencies of the form $Y_{:,j}=r\cdot f\left(X_{:,i}\right)+\sigma \cdot \varepsilon$, where r∈[0.5,1.0], σ=0.3, and *f* takes one of five functional forms: linear, quadratic, sinusoidal, piecewise, and cubic. We compared FastMI against Pearson and Spearman correlation on their ability to rank planted pairs above the 60×60−8=3592 independent pairs, using AUROC as the evaluation metric. Results were averaged over five random seeds.

As summarized in Table 10, FastMI attains a perfect AUROC of 1.000±0.000 across all five dependency types. In the most challenging sinusoidal case, the empirical Pearson AUROC falls to 0.492±0.178, close to chance, consistent with a weak linear association despite the planted nonlinear dependence. These results support the use of mutual-information-based estimators for capturing nonlinear cross-omics dependencies that linear methods may miss, and justify FastMI as the basis for heterogeneous-graph edge construction in FastMI-HGNet.

### 3.6. Omics Removal Ablation Study

**Figure 2:**
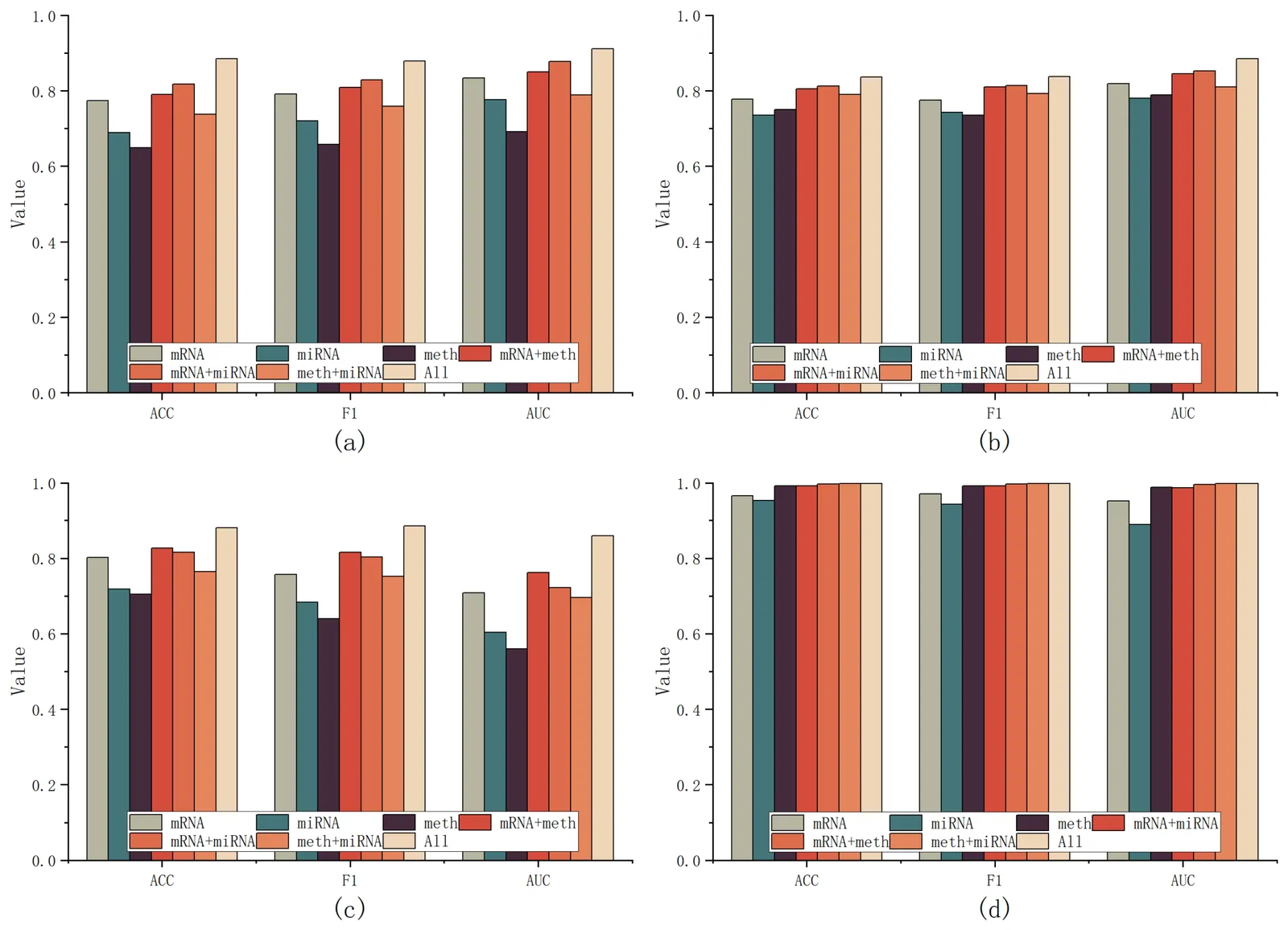
Omics removal ablation study results. (**a**,**b**) Performance across ROSMAP and LGG datasets; (**c,d**) Performance across BRCA and KIPAN datasets.

To analyze the contribution of each omics modality, we conduct an ablation study by systematically removing one or two omics layers and evaluating the model’s classification performance. The results are summarized in Figure 2, where subfigures (a) to (d) correspond to the results on ROSMAP, LGG, BRCA, and KIPAN datasets.

As shown in the figure, using a single omics modality (mRNA, miRNA, or DNA methylation) generally leads to lower classification performance across the four original benchmarks in terms of accuracy, F1-score, and AUC. While certain omics layers achieve relatively better performance in specific datasets, they often fail to capture the full complexity of disease subtypes.

When two omics layers are combined, model performance improves, highlighting the complementary nature of different molecular features. The highest classification accuracy and robustness are achieved when all three omics layers (mRNA + miRNA + DNA methylation) are included, suggesting that integrating multi-omics data provides a more comprehensive biological representation. This result supports the view that different molecular layers contribute complementary information for disease classification and subtype identification.

### 3.7. Biomarker Analysis

**Figure 3:**
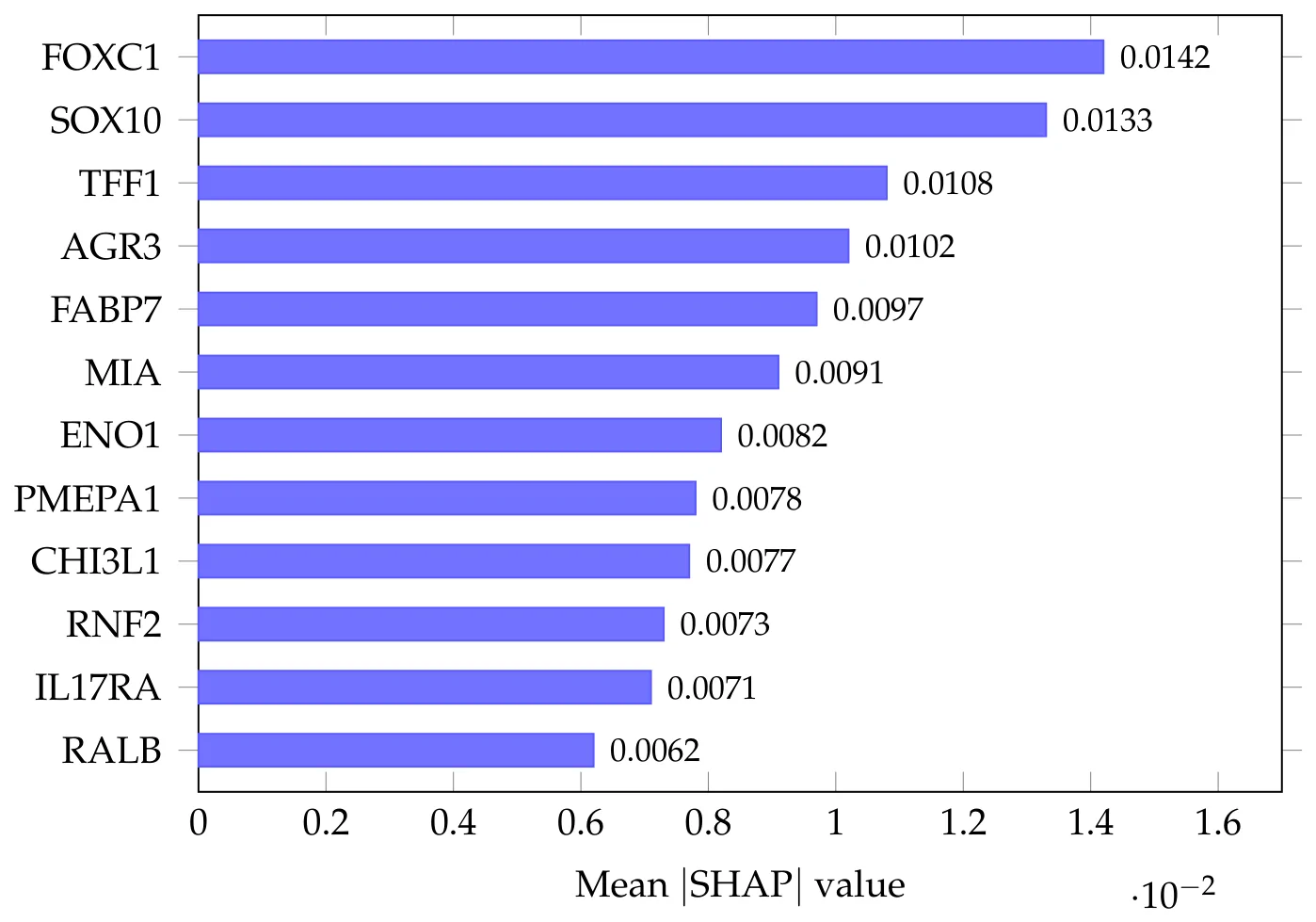
SHAP-based feature-importance ranking of the top-12 DNA-methylation-annotated genes for BRCA molecular-subtype classification. Bars show the mean absolute SHAP value averaged over the test samples and the five PAM50 output classes.

To identify the features driving the classification, we ranked all input features by their mean absolute SHAP value, averaged over the test samples and the five output classes. Figure 3 shows the twelve DNA-methylation-annotated genes with the highest attribution, among which FOXC1 and SOX10 received the largest weights, followed by TFF1 and AGR3. Corresponding SHAP-based feature-importance analyses for the mRNA and miRNA layers are provided in Supplementary Figures S1 and S2, respectively.

**Figure 4:**
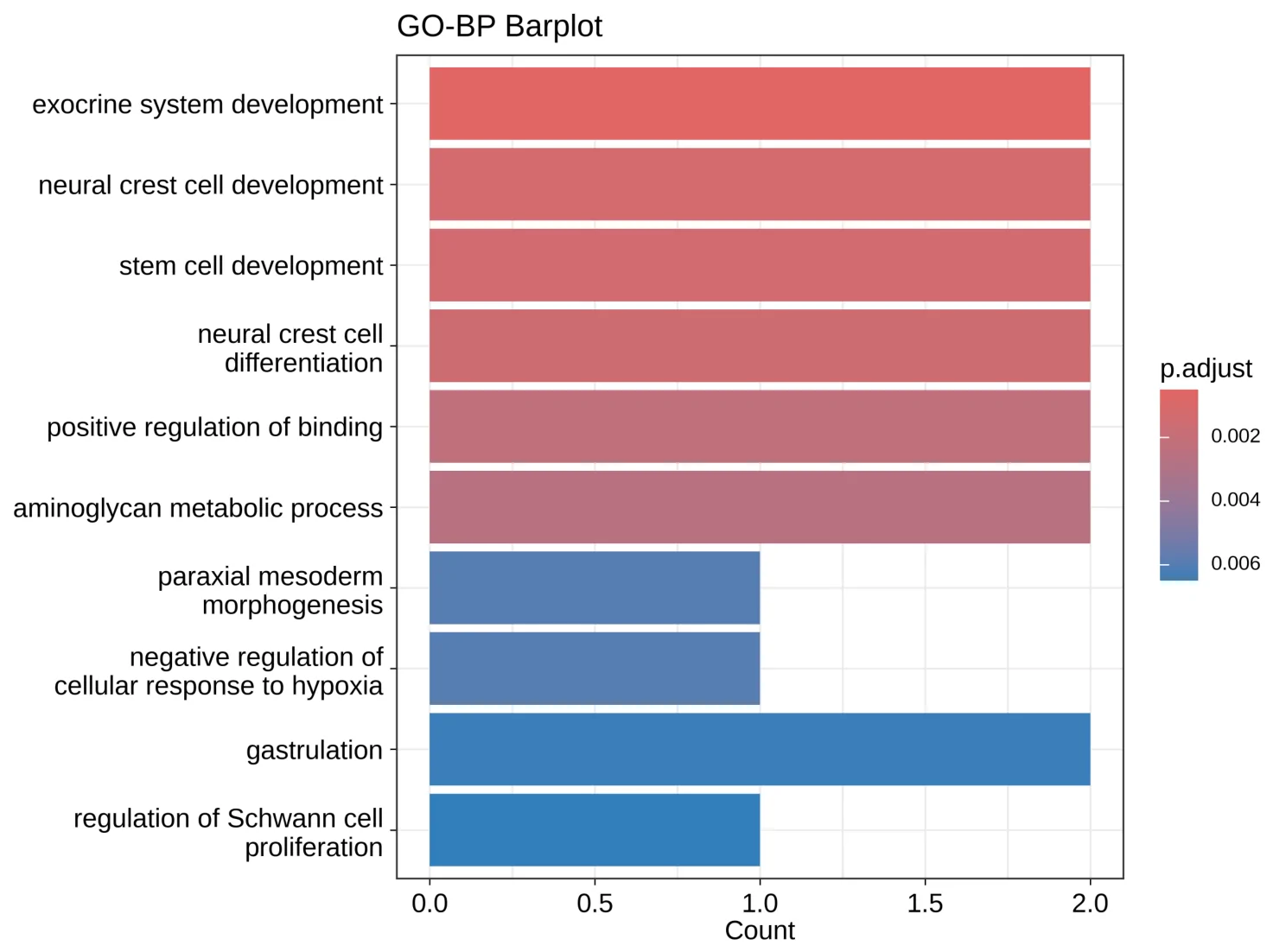
Gene Ontology (GO) Biological Process enrichment analysis of the top 12 SHAP-prioritized genes (DNA methylation layer) in the BRCA dataset. The bar plot shows GO terms passing the nominal enrichment threshold described in the Section 2. Bar color displays the multiple-testing-adjusted *p*-value for reference.

Here, BRCA denotes the TCGA breast cancer cohort (not the BRCA1/BRCA2 genes). We performed GO Biological Process enrichment analysis on the top 12 genes identified by SHAP from the DNA methylation layer. The threshold of 12 was determined by an elbow analysis of cumulative SHAP importance: beyond the 12th feature, the marginal contribution of each additional gene to total model attribution decreases to below 2%, indicating that the top-12 set accounts for a major portion of model attribution. Using the predefined nominal enrichment threshold described in the Section 2 (p<0.05), the analysis identified biological processes related to exocrine system development, stem cell development, immune response, and inflammatory regulation, as illustrated in Figure 4. These results provide exploratory biological context for the model-prioritized genes in BRCA molecular subtyping.

Several of these SHAP-prioritized genes are established breast-cancer subtype markers, supporting the biological plausibility of the model’s attributions. FOXC1 and SOX10 are well-characterized markers of the basal-like/triple-negative subtype [35,36], while FABP7 and MIA are likewise associated with basal/neural-crest-like phenotypes; in contrast, the estrogen-regulated TFF1 and the prognostic factor AGR3 [37] are markers of ER-positive/luminal disease. The remaining genes (ENO1, PMEPA1, RNF2, CHI3L1, RALB, and IL17RA) have documented roles in breast-cancer progression or the tumor microenvironment. The co-occurrence of markers spanning both the basal-like and luminal axes is consistent with the five-class PAM50 setting and indicates that the model prioritized subtype-relevant biology rather than arbitrary features. Because these variables are annotated at the gene-locus level of the DNA-methylation matrix, we regard them as candidate subtype-associated loci and do not infer CpG-specific regulatory mechanisms; the associations are therefore exploratory and hypothesis-generating.

**Figure 5:**
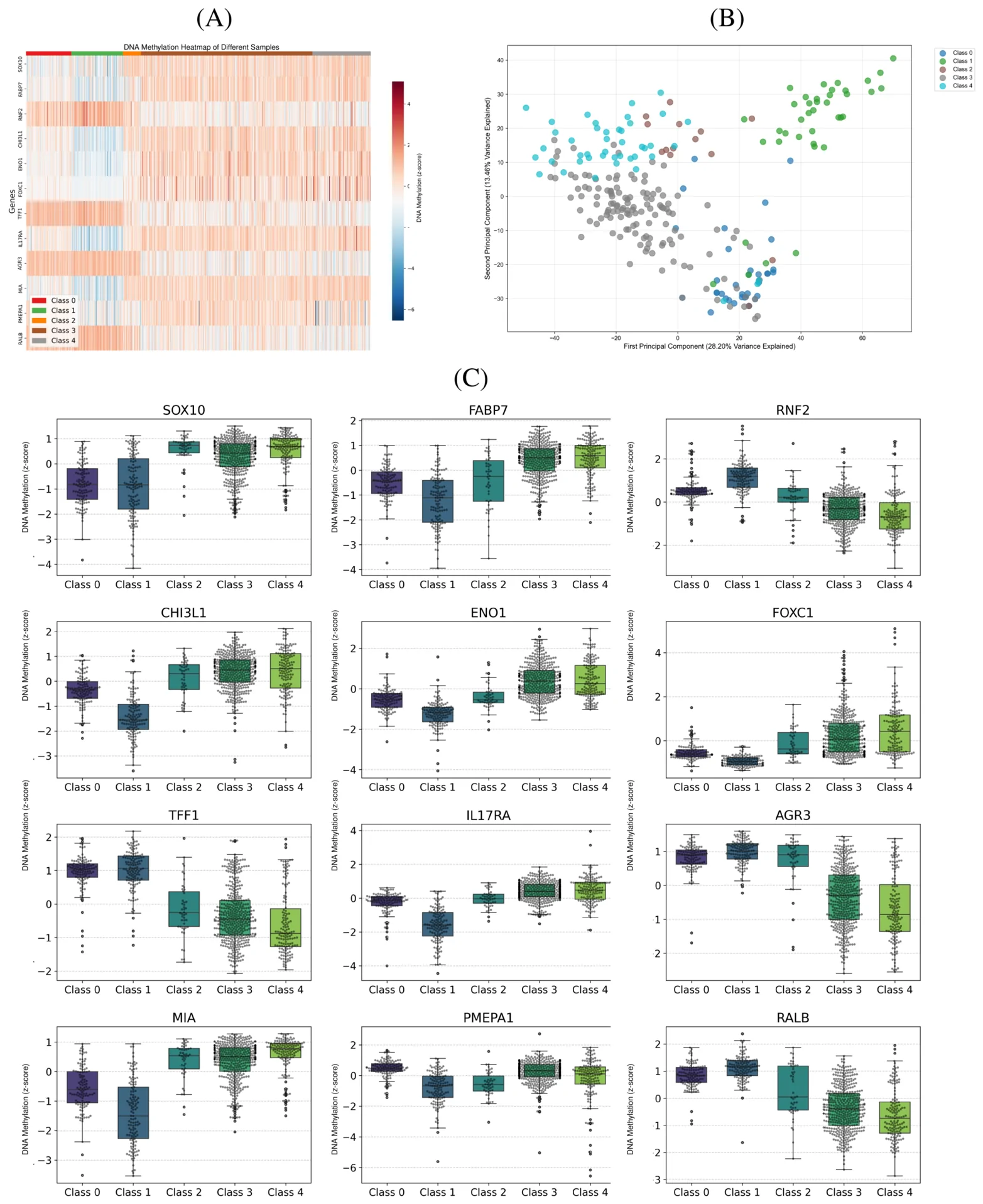
Complementary visualizations of molecular-subtype structure in the TCGA BRCA cohort. (**A**) Heatmap of standardized DNA methylation values for the 12 SHAP-prioritized features across all 875 samples grouped by molecular subtype. (**B**) Two-dimensional PCA projection of the 263 held-out test samples based on the concatenated preprocessed mRNA, DNA methylation, and miRNA features; PC1 and PC2 explain 28.20% and 13.46% of the variance, respectively. (**C**) DNA methylation distributions of the 12 selected features across all 875 samples, stratified by molecular subtype. Classes 0–4 denote Normal-like, Basal-like, HER2-enriched, Luminal A, and Luminal B, respectively. Each panel retains its original legend or class-axis labeling; colors should therefore be interpreted within each panel.

To further investigate these 12 methylation-prioritized features, we examined their standardized DNA methylation values across the five molecular subtypes in all 875 BRCA samples. Figure 5A presents a heatmap of the selected features, and Figure 5C shows their distributions by subtype. Both views reveal pronounced subtype-associated differences.

We also used principal component analysis to visualize the global subtype structure of the 263 held-out test samples based on their concatenated preprocessed mRNA, DNA methylation, and miRNA features. As shown in Figure 5B, samples from different classes exhibit distinct clustering patterns. The first and second principal components explain 28.20% and 13.46% of the variance, respectively.

To evaluate the statistical relevance of the selected features, we performed ANOVA tests to assess differences in their DNA methylation signals across the five molecular subtypes. Table 11 presents the test statistics and corresponding *p*-values for each feature. All 12 features show statistically significant differences across subtypes, supporting their relevance as model-prioritized subtype-associated loci.

These findings suggest that the selected DNA methylation features differ across molecular subtypes and may contribute to the model’s stratification of BRCA samples.

## 4. Discussion

In this study, we introduced FastMI-HGNet, a two-stream heterogeneous graph neural network for multi-omics disease classification. Evaluation on five public benchmarks (ROSMAP, LGG, KIPAN, BRCA, and COAD) showed that FastMI-HGNet achieved competitive performance compared with traditional machine learning and recent deep learning-based integration methods. The framework performed particularly well in the BRCA five-class molecular subtyping task, achieving an accuracy of 0.881 and a macro-F1 score of 0.859. On the more challenging COAD early- versus late-stage classification task, which lacks a performance ceiling, FastMI-HGNet remained competitive with the strongest deep-learning baselines while achieving the smallest standard deviation across all three metrics, indicating stable behavior in difficult, non-separable settings. These results suggest that combining vector-level feature modeling with FastMI-guided graph structure can help extract complementary information from multi-omics data.

The performance of FastMI-HGNet reflects three complementary design choices. First, FastMI-derived dependency edge priors guide the construction of a heterogeneous graph that captures nonlinear cross-omics dependency patterns beyond simple linear correlation. Second, the dual-stream architecture combines a Transformer-based data stream with a GAT-based graph stream, allowing the model to integrate vector-level feature interactions with graph-structured sample–feature and feature–feature relationships. Third, the uncertainty-aware ensemble provides a reliability metric for each prediction and helps identify ambiguous or low-confidence samples for further review.

When compared to state-of-the-art multi-omics integration methods, FastMI-HGNet provides a unified heterogeneous graph that explicitly models cross-layer interactions. For instance, graph-based methods like MOGONET typically construct omics-specific homogeneous graphs and fuse them at a later stage, which may fail to capture direct inter-omics feature relationships from the outset. In contrast, FastMI-HGNet introduces an explicit structural prior through FastMI-derived dependency edge priors, supporting interpretability and representation learning. The contribution of each component was assessed through ablation studies, where the removal of either the GNN stream, the Transformer stream, or the FastMI-derived dependency edge priors led to a discernible drop in performance. Among these components, removing the graph stream produced the largest performance drop across the four original benchmarks, identifying it as the most influential module, whereas the FastMI-derived edges provided the key structural signal within that stream (consistent with the hyperparameter sensitivity analysis, in which the FastMI threshold had the largest overall impact).

Beyond its classification accuracy, FastMI-HGNet offers biological interpretability through feature-level analysis. Through SHAP analysis on the BRCA dataset, we identified 12 gene-annotated features from the DNA methylation layer that were most influential to the model’s predictions. GO enrichment analysis linked these genes to processes including exocrine system development and immune-related regulation, and genes such as FOXC1 and SOX10 received high feature-importance scores. The selected methylation signals also differed across subtypes, supporting their relevance to molecular classification. These results support the use of FastMI-HGNet for model-based feature prioritization and hypothesis generation in BRCA subtyping.

Several limitations should be considered. First, the models were evaluated on publicly available retrospective datasets, and independent multi-center cohorts will be useful for assessing generalizability in future studies. Second, FastMI-derived edges summarize nonlinear dependency patterns rather than directional or intervention-level causal effects; perturbation experiments or prospective validation could further evaluate the biological mechanisms suggested by the model. Third, the current implementation integrates mRNA, miRNA, and DNA methylation data, and future work could incorporate proteomics, metabolomics, digital pathology, or curated pathway knowledge to improve biological coverage. Finally, some benchmarks, especially KIPAN, are highly separable because of strong molecular differences among renal cancer subtypes. Therefore, performance on more challenging tasks such as BRCA molecular subtyping and COAD stage classification should be emphasized when interpreting model utility. In addition, the local feature-mixing augmentation shuffles feature values across patients and may not preserve the biological covariance structure of transcriptomic profiles. Because this augmentation is confined to the training set, the validation and test samples remain unchanged, thereby avoiding direct contamination of the evaluation data. We nevertheless acknowledge that this perturbation may alter biological covariance, and future work could adopt label-preserving, within-subtype mixing to better preserve that structure.

Future work may incorporate prior biological knowledge—such as curated pathway annotations from KEGG/Reactome or protein-protein interaction networks—before graph construction, reducing reliance on purely data-driven feature selection. Future studies may also analyze the augmented multi-omics feature space in greater detail. For example, pathway-informed structured factor analysis or supervised UMAP could help separate primary subtype-associated variation from secondary sources such as technical batch effects or augmentation-induced perturbations.

In conclusion, FastMI-HGNet provides a FastMI-guided heterogeneous graph framework for multi-omics disease classification. By combining dependency edge priors, graph-based representation learning, a Transformer-based data stream, and uncertainty-aware ensemble learning, the framework integrates vector-level and graph-structured information from multiple omics layers. Evaluation on public benchmarks supports its utility for molecular classification and candidate biomarker prioritization. Future work will extend the framework to additional omics layers, independent cohorts, and prognostic or treatment-response prediction tasks.

## Figures and Tables

**Table 1:** Summary of the processed multi-omics benchmark datasets before training-set feature selection, showing sample distribution across categories and feature counts for each omics layer.

Dataset	Categories	Number of Features (mRNA, Methylation, miRNA)
ROSMAP	NC: 169, AD: 182	55,889, 23,788, 309
LGG	Grade 2: 246, Grade 3: 264	20,531, 20,114, 548
KIPAN	KICH: 66, KIRC: 318, KIRP: 274	20,531, 20,111, 445
BRCA	Normal-like: 115, Basal-like: 131, HER2-enriched: 46, Luminal A: 436, Luminal B: 147	20,531, 20,106, 503
COAD	Early stage: 155, Late stage: 121	20,531, 20,113, 1046

**Table 2:** Computational cost of FastMI-based dependency-edge construction and heterogeneous graph construction on a single NVIDIA A100 GPU. “Feature pairs” denotes the number of pairwise mutual-information computations.

Dataset	Features (*P*)	Feature Pairs	FastMI (min)	Graph (s)	Total (min)	Peak mem. (GiB)
ROSMAP	4309	9,281,586	3.13	0.88	3.15	0.20
LGG	4548	10,339,878	6.02	1.35	6.04	0.25
KIPAN	4445	9,876,790	8.44	1.71	8.47	0.27
BRCA	4503	10,136,253	19.63	2.29	19.67	0.34
COAD	5046	12,728,535	7.10	1.67	7.13	0.31
Total	—	52,363,042	44.32	7.90	44.45	—

**Table 5:** Classification results on the COAD early- vs. late-stage benchmark (mean ± standard deviation). F1 denotes the positive-class (late-stage) F1-score.

Method	ACC	F1	AUC
SVM [23]	0.584 ± 0.034	0.561 ± 0.041	0.612 ± 0.038
KNN [24]	0.539 ± 0.042	0.547 ± 0.045	0.561 ± 0.043
Lasso [25]	0.562 ± 0.029	0.583 ± 0.031	0.594 ± 0.035
RF [29]	0.591 ± 0.025	0.574 ± 0.028	0.643 ± 0.022
XGBoost [31]	0.608 ± 0.031	0.592 ± 0.036	0.657 ± 0.029
FCNN [32]	0.579 ± 0.037	0.601 ± 0.033	0.631 ± 0.041
BPLSDA [30]	0.583 ± 0.028	0.569 ± 0.030	0.626 ± 0.032
BSPLSDA [30]	0.551 ± 0.036	0.558 ± 0.039	0.582 ± 0.042
GRidge [33]	0.571 ± 0.027	0.595 ± 0.024	0.609 ± 0.031
CF [26]	0.594 ± 0.023	0.612 ± 0.021	0.669 ± 0.022
TMC [27]	0.623 ± 0.019	0.618 ± 0.017	0.684 ± 0.018
GMU [34]	0.611 ± 0.028	0.627 ± 0.026	0.672 ± 0.027
MOGONET [15]	0.604 ± 0.026	0.635 ± 0.024	0.691 ± 0.023
Dynamic [18]	0.629 ± 0.020	0.631 ± 0.018	0.698 ± 0.019
MLCLNet [19]	0.615 ± 0.025	0.642 ± 0.023	0.687 ± 0.022
CrossTrans [28]	0.626 ± 0.022	0.648 ± 0.020	0.708 ± 0.019
FastMI-HGNet	0.638 ± 0.016	0.655 ± 0.017	0.702 ± 0.016

**Table 6:** Ablation study results on binary classification tasks (ROSMAP, LGG).

Method	ROSMAP			LGG		
	ACC	F1	AUC	ACC	F1	AUC
FastMI-HGNet	0.885	0.879	0.911	0.836	0.838	0.885
-GNN	0.868	0.865	0.897	0.821	0.824	0.872
-Transformer	0.872	0.868	0.899	0.828	0.829	0.877
-FastMI	0.877	0.872	0.904	0.832	0.835	0.880
Best Baseline	0.865	0.863	0.912	0.835	0.840	0.886

**Table 7:** Ablation study results on multi-class classification tasks (BRCA, KIPAN).

Method	BRCA			KIPAN		
	ACC	F1_weighted_	F1_macro_	ACC	F1_weighted_	F1_macro_
FastMI-HGNet	0.881	0.885	0.859	0.999	0.999	0.999
-GNN	0.866	0.872	0.844	0.997	0.997	0.997
-Transformer	0.870	0.875	0.850	0.998	0.998	0.998
-FastMI	0.875	0.879	0.853	0.998	0.998	0.998
Best Baseline	0.877	0.880	0.853	0.999	0.999	0.999

**Table 8:** Sensitivity of test-set accuracy (ACC) to the three graph-construction thresholds. Each threshold was varied while the others were fixed at the values used in the main experiments; the main-experiment value in each group is shown in bold. Performance is stable within a broad range around these values, and the FastMI threshold $\tau_{\mathrm{FastMI}}$ has the largest overall impact.

Threshold	ROSMAP	LGG	KIPAN	BRCA	COAD
*FastMI dependency threshold * $\tau_{\mathrm{FastMI}}$					
0.1	0.873	0.828	0.998	0.872	0.626
0.2	0.881	0.835	0.999	0.879	0.634
**0.3**	**0.885**	**0.836**	**0.999**	**0.881**	**0.638**
0.4	0.880	0.837	0.999	0.878	0.631
0.5	0.876	0.830	0.998	0.874	0.625
*Sample–sample correlation threshold * $\tau_{\mathrm{sample}}$					
0.3	0.878	0.831	0.998	0.875	0.629
0.5	0.884	0.834	0.999	0.880	0.640
**0.7**	**0.885**	**0.836**	**0.999**	**0.881**	**0.638**
0.9	0.880	0.838	0.998	0.879	0.633
*Sample–feature threshold* $\tau_{\mathrm{feat}}$ (|z|; two-sided retention)					
1.0 (∼31.7%)	0.876	0.831	0.998	0.874	0.628
1.5 (∼13.4%)	0.883	0.837	0.999	0.880	0.639
**2.0** (∼4.6%)	**0.885**	**0.836**	**0.999**	**0.881**	**0.638**
2.5 (∼1.2%)	0.879	0.832	0.998	0.875	0.630

**Table 9:** Bootstrap 95% confidence intervals (1000 resamples) for FastMI-HGNet on the primary metric of each benchmark, together with paired significance tests against a classical baseline (SVM) and a recent high-performing comparator (DeLong test for AUC; paired bootstrap for F1_macro_). n.s.: not significant (p>0.05).

Dataset	Metric	FastMI-HGNet (95% CI)	vs. SVM	vs. Recent Comparator
ROSMAP	AUC	0.911 (0.860–0.962)	p<0.001	Dynamic, 0.912; n.s. (p=0.85)
LGG	AUC	0.885 (0.858–0.912)	p<0.001	MLCLNet, 0.886; n.s. (p=0.90)
BRCA	F1_macro_	0.859 (0.821–0.897)	p<0.001	CrossTrans, 0.853; n.s. (p=0.44)
KIPAN	F1_macro_	0.999 (0.994–1.000)	n.s. (p=0.29)	TMC, 0.994; n.s. (p=0.97)
COAD	AUC	0.702 (0.612–0.803)	p<0.01	CrossTrans, 0.708; n.s. (p=0.74)

**Table 10:** Cross-omics dependency recovery (AUROC, mean ± s.d. over 5 seeds) under five planted dependency types (K=8 planted vs. 3592 independent feature pairs; $X,Y\in R^{200\times 60}$). FastMI attains perfect ranking across all settings, while Pearson and Spearman degrade markedly on sinusoidal dependencies.

Dependency Type	FastMI	Pearson	Spearman
Linear	1.000±0.000	1.000±0.000	1.000±0.000
Quadratic	1.000±0.000	0.732±0.096	0.644±0.086
Sinusoidal	1.000±0.000	0.492±0.178	0.754±0.093
Piecewise	1.000±0.000	1.000±0.000	1.000±0.000
Cubic	1.000±0.000	1.000±0.000	1.000±0.000
Mean (5 types)	1.000±0.000	0.845±0.225	0.880±0.163

**Table 11:** ANOVA results for the 12 selected DNA methylation features across the five BRCA molecular subtypes.

Feature	Test Statistic	*p*-Value
SOX10	87.5241	$1.62\times 10^{-62}$
FABP7	135.0213	$9.85\times 10^{-90}$
RNF2	113.0477	$1.27\times 10^{-77}$
CHI3L1	155.3584	$2.71\times 10^{-100}$
ENO1	141.5079	$3.64\times 10^{-93}$
FOXC1	69.6761	$3.37\times 10^{-51}$
TFF1	135.0176	$9.89\times 10^{-90}$
IL17RA	232.7039	$8.23\times 10^{-136}$
AGR3	139.1669	$6.20\times 10^{-92}$
MIA	184.8508	$1.24\times 10^{-114}$
PMEPA1	48.2477	$1.13\times 10^{-36}$
RALB	151.7475	$1.85\times 10^{-98}$

## Data Availability

The TCGA-derived datasets analyzed in this study (LGG, KIPAN, and BRCA) are publicly available through the GDC Data Portal at https://portal.gdc.cancer.gov (accessed on 24 June 2026). The ROSMAP dataset is available through Synapse at https://www.synapse.org/Synapse:syn3219045 (accessed on 24 June 2026), subject to the corresponding data-access requirements. The batch-effect-normalized TCGA colon adenocarcinoma (COAD) multi-omics and clinical data used for the early- versus late-stage benchmark were obtained from the UCSC Xena platform at https://xenabrowser.net (accessed on 24 June 2026). The processed multi-omics benchmark matrices used in this study follow the data format described by Wang et al. for MOGONET, as detailed in the Section 2. The implementation of FastMI-HGNet is available from the corresponding author upon reasonable request.

## Supplementary Materials

The following supporting information can be downloaded at https://www.mdpi.com/article/10.3390/genes17080865/s1, Figure S1: SHAP-based feature-importance analysis of the mRNA layer in the TCGA breast cancer (BRCA) cohort; Figure S2: SHAP-based feature-importance analysis of the miRNA layer in the TCGA BRCA cohort.

## References

[1] Chen C., Wang J., Pan D., Wang X., Xu Y., Yan J., Wang L., Yang X., Yang M., Liu G. (2023). Applications of Multi-omics Analysis in Human Diseases. MedComm.

[2] Huang S., Chaudhary K., Garmire L.X. (2017). More is better: Recent progress in multi-omics data integration methods. Front. Genet..

[3] Michaut M., Chin S.F., Majewski I., Severson T.M., Bismeijer T., De Koning L., Peeters J.K., Schouten P.C., Rueda O.M., Bosma A.J. (2016). Integration of genomic, transcriptomic and proteomic data identifies two biologically distinct subtypes of invasive lobular breast cancer. Sci. Rep..

[4] Hasin Y., Seldin M., Lusis A. (2017). Multi-omics approaches to disease. Genome Biol..

[5] Chaudhary K., Poirion O.B., Lu L., Garmire L.X. (2018). Deep learning–based multi-omics integration robustly predicts survival in liver cancer. Clin. Cancer Res..

[6] Lin Y., Zhang W., Cao H., Li G., Du W. (2020). Classifying breast cancer subtypes using deep neural networks based on multi-omics data. Genes.

[7] Poirion O.B., Jing Z., Chaudhary K., Huang S., Garmire L.X. (2021). DeepProg: An ensemble of deep-learning and machine-learning models for prognosis prediction using multi-omics data. Genome Med..

[8] Way G.P., Greene C.S. (2018). Extracting a biologically relevant latent space from cancer transcriptomes with variational autoencoders. Pac. Symp. Biocomput..

[9] Sun Q., Cheng L., Meng A., Ge S., Chen J., Zhang L., Gong P. (2023). SADLN: Self-attention based deep learning network of integrating multi-omics data for cancer subtype recognition. Front. Genet..

[10] Liu Z., Park T. (2024). DMOIT: Denoised multi-omics integration approach based on transformer multi-head self-attention mechanism. Front. Genet..

[11] Lan W., Liao H., Chen Q., Zhu L., Pan Y., Chen Y.P.P. (2024). DeepKEGG: A multi-omics data integration framework with biological insights for cancer recurrence prediction and biomarker discovery. Brief. Bioinform..

[12] Moon S., Lee H. (2022). MOMA: A multi-task attention learning algorithm for multi-omics data interpretation and classification. Bioinformatics.

[13] Wang B., Mezlini A.M., Demir F., Fiume M., Tu Z., Brudno M., Haibe-Kains B., Goldenberg A. (2014). Similarity network fusion for aggregating data types on a genomic scale. Nat. Methods.

[14] Kipf T.N., Welling M. (2016). Semi-supervised classification with graph convolutional networks. arXiv.

[15] Wang T., Shao W., Huang Z., Tang H., Zhang J., Ding Z., Huang K. (2021). MOGONET integrates multi-omics data using graph convolutional networks allowing patient classification and biomarker identification. Nat. Commun..

[16] Li X., Ma J., Leng L., Han M., Li M., He F., Zhu Y. (2022). MoGCN: A multi-omics integration method based on graph convolutional network for cancer subtype analysis. Front. Genet..

[17] Subramanian I., Verma S., Kumar S., Jere A., Anamika K. (2020). Multi-omics data integration, interpretation, and its application. Bioinform. Biol. Insights.

[18] Han Z., Yang F., Huang J., Zhang C., Yao J. (2022). Multimodal dynamics: Dynamical fusion for trustworthy multimodal classification. Proceedings of the IEEE/CVF Conference on Computer Vision and Pattern Recognition.

[19] Zheng X., Tang C., Wan Z., Hu C., Zhang W. (2023). Multi-level confidence learning for trustworthy multimodal classification. Proceedings of the AAAI Conference on Artificial Intelligence.

[20] Goldman M.J., Craft B., Hastie M., Repečka K., McDade F., Kamath A., Banerjee A., Luo Y., Rogers D., Brooks A.N. (2020). Visualizing and interpreting cancer genomics data via the Xena platform. Nat. Biotechnol..

[21] Rahimi A., Gönen M. (2018). Discriminating early- and late-stage cancers using multiple kernel learning on gene sets. Bioinformatics.

[22] Kraskov A., Stögbauer H., Grassberger P. (2004). Estimating mutual information. Phys. Rev. E Stat. Nonlinear Soft Matter Phys..

[23] Lanckriet G.R., De Bie T., Cristianini N., Jordan M.I., Noble W.S. (2004). A statistical framework for genomic data fusion. Bioinformatics.

[24] Fix E., Hodges J.L. (1951). Discriminatory Analysis—Nonparametric Discrimination: Consistency Properties.

[25] Ranstam J., Cook J.A. (2018). LASSO regression. J. Br. Surg..

[26] Hong D., Gao L., Yokoya N., Yao J., Chanussot J., Du Q., Zhang B. (2020). More diverse means better: Multimodal deep learning meets remote-sensing imagery classification. IEEE Trans. Geosci. Remote Sens..

[27] Han Z., Zhang C., Fu H., Zhou J.T. (2022). Trusted multi-view classification with dynamic evidential fusion. IEEE Trans. Pattern Anal. Mach. Intell..

[28] Xie W., Fang Y., Yang G., Yu K., Li W. (2023). Transformer-based multi-modal data fusion method for COPD classification and physiological and biochemical indicators identification. Biomolecules.

[29] Ho T.K. (1995). Random decision forests. Proceedings of the 3rd International Conference on Document Analysis and Recognition.

[30] Singh A., Shannon C.P., Gautier B., Rohart F., Vacher M., Tebbutt S.J., Lê Cao K.A. (2019). DIABLO: An integrative approach for identifying key molecular drivers from multi-omics assays. Bioinformatics.

[31] Chen T., Guestrin C. (2016). Xgboost: A scalable tree boosting system. Proceedings of the 22nd ACM SIGKDD International Conference on Knowledge Discovery and Data Mining.

[32] Chang Y., Park H., Yang H.J., Lee S., Lee K.Y., Kim T.S., Jung J., Shin J.M. (2018). Cancer drug response profile scan (CDRscan): A deep learning model that predicts drug effectiveness from cancer genomic signature. Sci. Rep..

[33] Van De Wiel M.A., Lien T.G., Verlaat W., van Wieringen W.N., Wilting S.M. (2016). Better prediction by use of co-data: Adaptive group-regularized ridge regression. Stat. Med..

[34] Arevalo J., Solorio T., Montes-y Gomez M., González F.A. (2020). Gated multimodal networks. Neural Comput. Appl..

[35] Ray P.S., Wang J., Qu Y., Sim M.-S., Shamonki J., Bagaria S.P., Ye X., Liu B., Elashoff D., Hoon D.S. (2010). FOXC1 is a potential prognostic biomarker with functional significance in basal-like breast cancer. Cancer Res..

[36] Cimino-Mathews A., Subhawong A.P., Elwood H., Warzecha H.N., Sharma R., Park B.H., Taube J.M., Illei P.B., Argani P. (2013). Neural crest transcription factor Sox10 is preferentially expressed in triple-negative and metaplastic breast carcinomas. Hum. Pathol..

[37] Garczyk S., von Stillfried S., Antonopoulos W., Hartmann A., Schrauder M.G., Fasching P.A., Anzeneder T., Tannapfel A., Ergönenc Y., Knüchel R. (2015). AGR3 in breast cancer: Prognostic impact and suitable serum-based biomarker for early cancer detection. PLoS ONE.

