# Supplementary material for: FastMI-HGNet: A Two-Stream Heterogeneous Graph Neural Network for Multi-Omics Disease Classification
Source: Genes (Basel). 2026 Jul 24;17(8):865. doi: 10.3390/genes17080865 (PMC13512668; doi:10.3390/genes17080865)
Supplement: Supplementary file 1 [file genes-17-00865-s001.zip › genes-4436990-supplementary.pdf]

Supplementary Material

FastMI-HGNet: A Two-Stream Heterogeneous Graph Neural Network for Multi-Omics Disease Classification

Throughout, "BRCA" denotes the TCGA breast cancer cohort (not the BRCA1/BRCA2 genes). In the main text, the top-12 SHAP-prioritized genes are derived from the **DNA methylation** layer. Figures S1 and S2 report the SHAP-based feature-importance rankings for the remaining omics layers of the BRCA cohort (mRNA and miRNA), complementing the DNA-methylation analysis presented in the main text. Bars show the mean absolute SHAP value averaged over the test samples and the five PAM50 output classes.

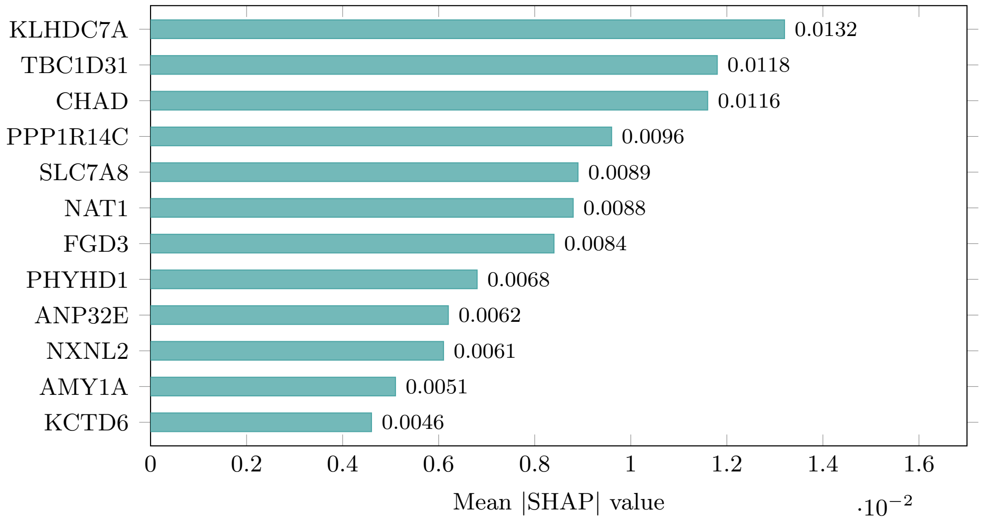

Figure S1: SHAP-based feature-importance ranking of the top-12 **mRNA** features in the TCGA BRCA cohort. TBC1D31 corresponds to the matrix identifier WDR67. Bars show the mean absolute SHAP value averaged over the test samples and the five PAM50 classes.

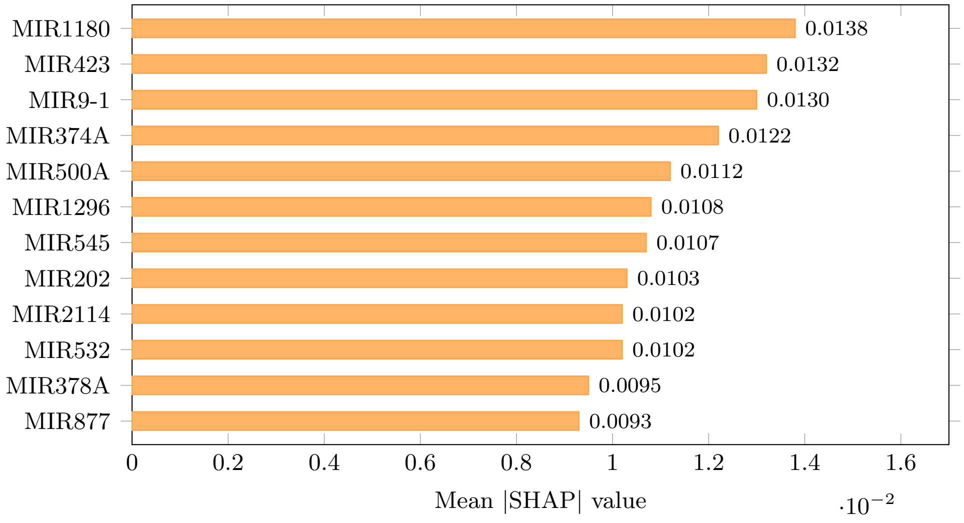

Figure S2: SHAP-based feature-importance ranking of the top-12 **miRNA** precursor-locus features in the TCGA BRCA cohort. Bars show the mean absolute SHAP value averaged over the test samples and the five PAM50 classes.
